# Applying the Electronic Health Literacy Lens: Systematic Review of Electronic Health Interventions Targeted at Socially Disadvantaged Groups

**DOI:** 10.2196/18476

**Published:** 2020-08-13

**Authors:** Christina Cheng, Alison Beauchamp, Gerald R Elsworth, Richard H Osborne

**Affiliations:** 1 Centre for Global Health and Equity Faculty of Health, Arts and Design Swinburne University of Technology Hawthorn Australia; 2 Deakin University School of Health and Social Development Faculty of Health Burwood Australia; 3 Department of Rural Health Monash University Melbourne Australia; 4 Department of Medicine - Western Health The University of Melbourne Melbourne Australia; 5 Australian Institute for Musculoskeletal Science Sunshine Hospital St Albans Australia

**Keywords:** eHealth, health literacy, internet, health care, telecommunications, digital divide, health equity

## Abstract

**Background:**

Electronic health (eHealth) has the potential to improve health outcomes. However, eHealth systems need to match the eHealth literacy needs of users to be equitably adopted. Socially disadvantaged groups have lower access and skills to use technologies and are at risk of being digitally marginalized, leading to the potential widening of health disparities.

**Objective:**

This systematic review aims to explore the role of eHealth literacy and user involvement in developing eHealth interventions targeted at socially disadvantaged groups.

**Methods:**

A systematic search was conducted across 10 databases for eHealth interventions targeted at older adults, ethnic minority groups, low-income groups, low-literacy groups, and rural communities. The eHealth Literacy Framework was used to examine the eHealth literacy components of reviewed interventions. The results were analyzed using narrative synthesis.

**Results:**

A total of 51 studies reporting on the results of 48 interventions were evaluated. Most studies were targeted at older adults and ethnic minorities, with only 2 studies focusing on low-literacy groups. eHealth literacy was not considered in the development of any of the studies, and no eHealth literacy assessment was conducted. User involvement in designing interventions was limited, and eHealth intervention developmental frameworks were rarely used. Strategies to assist users in engaging with technical systems were seldom included in the interventions, and accessibility features were limited. The results of the included studies also provided inconclusive evidence on the effectiveness of eHealth interventions.

**Conclusions:**

The findings highlight that eHealth literacy is generally overlooked in developing eHealth interventions targeted at socially disadvantaged groups, whereas evidence about the effectiveness of such interventions is limited. To ensure equal access and inclusiveness in the age of eHealth, eHealth literacy of disadvantaged groups needs to be addressed to help avoid a digital divide. This will assist the realization of recent technological advancements and, importantly, improve health equity.

## Introduction

### Background

Electronic health (eHealth), “the use of information and communications technology (ICT) in support of health and health-related fields” [1], is increasingly being integrated into the delivery of health resources and services. The World Health Organization (WHO) [2] also recognizes that digital technologies have the potential to accelerate toward achieving Sustainable Development Goals by improving health services. However, not everyone has substantive ICT access or skills to take advantage of the benefits of eHealth.

The issue of inequitable access, usage or skills, and outcomes relating to ICT by subgroups of society, described as the *digital divide* [3-6], is a recognized public health concern [7]. The sociodemographic factors associated with health disparities, such as age, income, education, and ethnicity, are similar to the characteristics of people who have limited ICT access or skills [8-10]. Older age, less education, lower income, being from an ethnic minority group, or living in a remote area are all associated with decreased access or less use of the internet for activities such as health information seeking, communicating with health care providers, monitoring health, or using personal health records [11-15]. As such, these socially disadvantaged groups are usually overlooked in eHealth design [15] and are at risk of becoming digitally marginalized [7,16], leading to a potential widening of health disparities.

In recognition of the different sets of skills required for using eHealth, the concept of eHealth literacy, defined as “the ability to seek, find, understand, and appraise health information from electronic sources and apply the knowledge gained to addressing or solving a health problem,” was introduced in 2006 [17]. This concept is grounded in health literacy [17,18], which is recognized as a critical determinant of health [19]. The concept of eHealth literacy has since been considered amid the everchanging landscape of ICT, and there is also a growing recognition that eHealth strategies will be ineffective and inequitable if the eHealth literacy needs of users are not addressed [20-22]. In 2015, Norgaard et al [23] developed the eHealth Literacy Framework (eHLF) by integrating the perspectives and experiences of a wide range of eHealth stakeholders, and 7 domains of eHealth literacy were identified. On the basis of this framework and applying a validity-driven approach to scale development [24], the eHealth Literacy Questionnaire (eHLQ) was also developed and tested [25]. The 7 domains of eHealth literacy are as follows: (1) using technology to process health information, (2) understanding of health concepts and language, (3) ability to actively engage with digital services, (4) feel safe and in control, (5) motivated to engage with digital services, (6) access to digital services that work and (7) digital services that suit individual needs.

According to the eHLF, eHealth literacy is not only the ability of an individual user but also relates to the system and how the two interact. For an eHealth intervention to be adopted, the system needs to align with the eHealth literacy needs of target users [23], which may differ across settings and contexts [25]. By assessing the eHealth literacy of target users, weaknesses in certain domains of eHealth literacy can be identified, and interventions can be designed to respond to the relevant weaknesses [23].

In reviewing the evaluation of the now defunct UK web-based personal health record HealthSpace, Monkman and Kushniruk [26] commented that the system did not match the eHealth literacy or information needs of users. Apart from the consideration of literacy, the evaluation also recommended that user-centered principles, such as involving users in design and development [27-29], be applied in any future endeavors [30].

### Objectives

eHealth literacy plays an important role in improving health outcomes across the socioeconomic spectrum. This systematic review aimed to apply an eHealth literacy lens to explore current practices in the development of eHealth interventions targeting socially disadvantaged groups, who are at risk of being digitally marginalized. Guided by the eHLF, this review examined not only the usability of eHealth interventions but also how interventions motivate users or address privacy concerns as part of the effort to respond to eHealth literacy needs. With the WHO recognizing health literacy as having the potential to empower and drive equity [19], insights into how interventions meet the needs of disadvantaged groups will highlight gaps in research and advance the role of eHealth literacy in making eHealth more accessible. The purpose of this review was to answer the following research questions:

1. Was eHealth literacy considered during the development of eHealth interventions targeted at socially disadvantaged groups? If yes, what approaches were used to determine the eHealth literacy needs of the target group?
2. What frameworks or theories were used to guide the development of eHealth interventions besides theories on eHealth literacy?
3. Were users involved in the development of eHealth interventions?
4. What eHealth literacy domains, as described in the eHLF, were likely addressed in the identified eHealth interventions?
5. Were eHealth interventions targeted at socially disadvantaged groups effective when eHealth literacy was considered?

## Methods

### Review Design

This systematic review followed the Preferred Reporting Items for Systematic Reviews and Meta-Analysis Protocols 2015 checklist [31]. This was a review with no patient or public involvement.

### Eligibility Criteria

The development of the inclusion criteria was based on the *PICO* (population, intervention, control, and outcomes) model [32]. The population referred to socially disadvantaged groups with any health condition, who were disadvantaged because of age, education, migrant status, living in a rural or remote area, or socioeconomic status [33]. For age, older adults were defined as people aged 60 years or older [34]. An intervention referred to eHealth interventions, systems, or applications mainly delivered through the internet via ICT such as computers, tablets, or mobile phones, targeted and operated by individual participants through platforms such as websites, apps, social media, email, or text messaging [35]. The interventions were those aimed at improving health or preventing or reducing the risk of illness. Study design included randomized controlled trials (RCTs) and non-RCT studies. Outcomes included clinical health outcomes or health knowledge and behavior. Only studies published in English peer-reviewed journals with full text available were included. Publication dates of studies were from January 2007 to July 2019. January 2007 was chosen because the concept of eHealth literacy was first introduced in late 2006 [17].

Studies were excluded if they were protocols, literature or systematic reviews, and studies of nonhealth outcomes, such as feasibility studies, usability evaluations, or economic evaluations. Studies of telehealth or telemedicine for monitoring physical conditions or medications that required no active participation from participants or only for communication with carers and health professionals were excluded. In addition, studies of consultations via videoconferencing, eHealth initiatives for risk assessment of physical conditions or motor- or cognitive skills training, or computer skills training and eHealth programs targeted at health care providers or carers were excluded. In cases where studies based on the same intervention with similar outcome measures were identified, any pilot studies of that intervention were excluded.

### Search Strategy and Study Selection

Studies were identified from 10 databases, including Academic Search Complete, AgeLine, Cumulative Index to Nursing and Allied Health Literature (CINAHL) Complete, Communication & Mass Media Complete, Excerpta Medica dataBASE (EMBASE), Education Resources Information Center (ERIC), Global Health, Medical Literature Analysis and Retrieval System Online (MEDLINE) Complete, American Psychological Association PsycInfo database (PsycINFO), and Sociology Research Database (SocINDEX), with searches conducted in November 2018 and updated in July 2019. Search terms were based on keywords from the inclusion criteria (Multimedia Appendix 1). The reference lists of relevant studies were also scanned for potential studies. The search and screening of titles and abstracts were conducted by one author (CC), who also reviewed the full text of potential studies with reasons for exclusion documented.

### Data Extraction and Quality Assessment

Following study selection, data were extracted based on the research questions, and study quality was appraised using the Effective Public Health Practice Project Quality Assessment Tool (Multimedia Appendix 2) [36-87]. The tool is considered a valid and reliable instrument, adaptable to most public health systematic reviews for evaluating a range of study designs [88,89]. Data extraction and quality assessment of 10% (6/51) of the included studies were independently reviewed by 2 authors (AB and CC). Discrepancies were resolved through discussion and consensus. Decisions from the discussion were used to guide the data extraction and the quality assessment of the remaining studies undertaken by one author (CC).

### Data Analysis

Owing to the heterogeneity of study designs and outcome measures among the included studies, a narrative synthesis was used to answer the research questions. For the research question relating to whether eHealth literacy domains were likely addressed in interventions, a directed content analysis approach was adopted. This approach allows researchers to use an existing theory or framework as coding categories, to develop operational definitions for each category as determined by the theory or framework, and to analyze the content accordingly [90]. For this review, the eHLF was used to code the eHealth literacy domains. The intervention components likely addressing each eHealth literacy domain were based on components derived from the concept mapping workshops used to develop the framework [23,25] and matched with the description of the intervention in the included studies. For example, the use of passwords to access the system or intervention is expected to promote a sense of security. Hence, the feature is coded as meeting the needs of *Domain 4 Feel safe and in control*. Providing information in users’ preferred language for interventions that target ethnic minorities will be a component that matches *Domain 7 Digital services that suit individual needs*. The classification of intervention components was initially undertaken by one author (CC), followed by discussion and review with one of the eHLF developers (RO) and among the other authors. The details of the intervention components relating to the eHealth literacy domains are presented in Table 1 [23]. The coding of 10% (6/51) of the studies was independently conducted by 2 authors (AB and CC). Discrepancies were resolved by discussion and consensus. Decisions following discussion were used to guide the coding of the remaining studies undertaken by 1 author (CC).

For the research question regarding the effectiveness of interventions, the overall effect size could not be determined because of the diversity of outcome measures and data analysis methods. Therefore, effectiveness was estimated by reporting statistically significant improvement between intervention and control groups or between before and after intervention for one-group pretest-posttest for the outcome measures stipulated. If more than 1 primary outcome measure was stated, only the first 3 were included.

**Table 1:** Examples of intervention components that likely address electronic health literacy domains derived from the eHealth Literacy Framework.

Descriptions		Examples of intervention components
**1. Using technology to process health information**		
	Able to read, write, and remember; apply basic numerical concepts; and understand context-specific language (such as health, technology, and English) as well as critically appraise information. Know when, how, and what information to use	Contains information about health conditions Contains health information in a format that can easily be understood (such as text in low reading grade, video, graphics, animations, graphs, stories, examples, culturally or locally relevant materials) Contains information that can help make decisions Can use the system to share information with family, friends, and health professionals Can use the system to organize or record personal health information (such as recording or monitoring activities, journal, diary, worksheets) Provides access to other information resources
**2. Understanding of health concepts and language**		
	Know about basic physiological functions and own current health status. Aware of risk factors and how to avoid them or reduce their influence on own health	Contains information that one can take responsibility for one’s own health (such as setting personal goals or plans, monitoring health, practical skills or tips, practical and usable information such as recipes, activities or opportunities to join events, and download information) Tailored information, instructions or personal guidance, and chat sessions Homework assignments or tests of knowledge or evaluation Provide easy-to-use tools for measurements or assessment or monitoring
**3. Ability to actively engage with digital services**		
	Being comfortable using digital services for handling information	Easy navigation around the system Detailed and easy-to-understand instructions Provide training or a manual to use the system
**4. Feel safe and in control**		
	Feel that they have the ownership of personal data stored in the system and that their data are safe and can be accessed only by people to whom the data are relevant (such as own doctor and nurse)	Unique username and password protected Secure website or database or communication Provide means to ensure privacy *Closed* system to which only authorized personnel have access Can maintain anonymity if needed
**5. Motivated to engage with digital services**		
	Feel that engaging in the use of digital services will be useful for them in managing their health	Incentives to return to use the systems Encouragement to continue to use the systems Alerts and notifications Quick response to queries Provides tailored feedback, progress reports, or support Provides new content regularly Regular meetup sessions or discussion forums Provides peer or professional support Quick and easy communication (such as sending or receiving emails, asking questions, and inquiries)
**6. Access to digital services that work**		
	Have access to digital services that the users trust to be working when they need it and as they expect it to work	Provides access to the hardware or system Provides technical support Can be accessed anytime anywhere Access to tools or devices that can be integrated into the system
**7. Digital services that suit individual needs**		
	Have access to digital services that suit the specific needs and preferences of the users. This includes responsive features of both the information technology and health care system as well as adaptation of devices and interfaces to be used by people with physical and mental disabilities	Consists of accessibility features such as change of font size or audio function Easy to use, efficient, and user-friendly interface (such as large buttons and large icon) Available in users’ preferred language

## Results

### Selection of Studies

**Figure 1:**
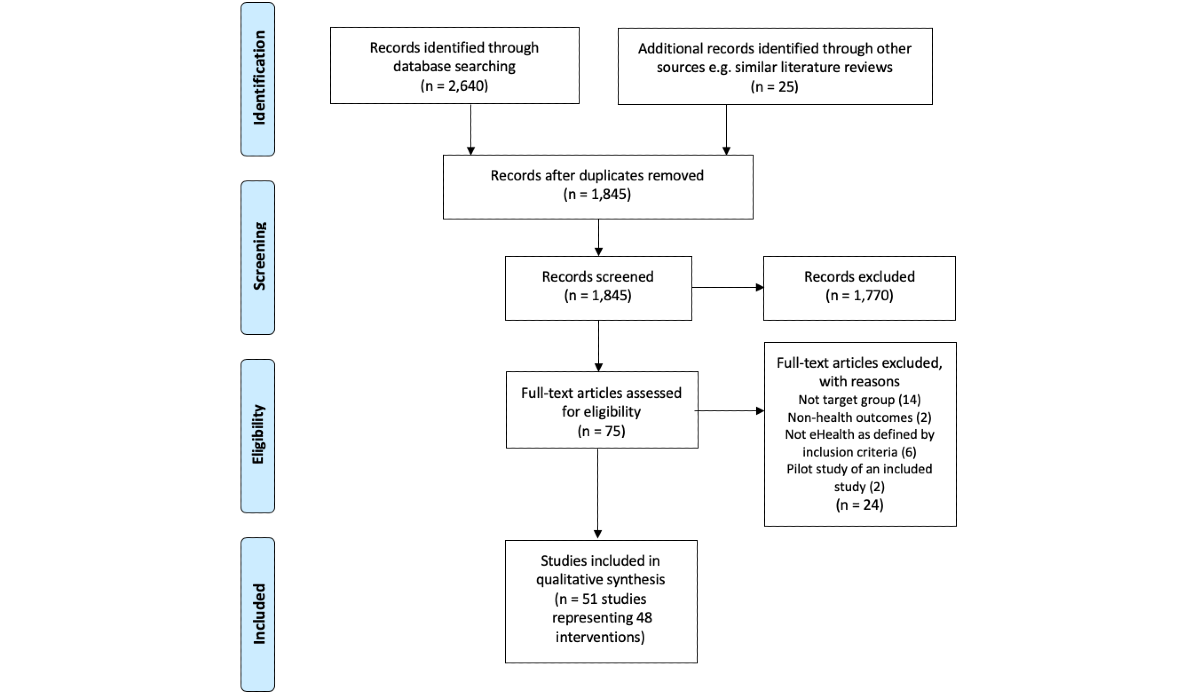
Selection process using the PRISMA Flow Diagram [31].

The search resulted in 2640 studies; after removing 820 duplicates and an additional 25 records identified through other sources, a total of 1845 records were screened. Following a review of titles and abstracts, 75 studies were retrieved for full text review. A total of 24 studies were excluded for reasons including non–target groups, non–health outcomes, and not eHealth as defined by the inclusion criteria and 2 pilot studies of included studies, resulting in 51 studies reporting on the findings of 48 interventions (Figure 1).

### Characteristics of the Studies

Among the 51 studies, 11 were pilot studies, 43 were RCTs (40 were two-armed RCTs, 2 were three-armed RCTs, and 1 was a five-armed RCT). A total of 7 studies used the one-group pretest-posttest design, and 1 was a case study. Among the 43 RCTs, 42 control groups received no intervention, waitlist, standard care, usual care, or in-person education, whereas 1 study did not describe this. The sample size ranged from 1 to 755 (Table 2).

Apart from the postintervention assessment, 13 studies also conducted follow-up assessments, ranging from 1 week to 12 months. Clinical health outcomes were reported in 22 studies, whereas 28 studies measured health-related outcomes such as attitude, behaviors, or knowledge, and 1 study measured both behavior and clinical outcomes. For the quality rating, 2 studies were rated as strong, 24 studies were rated as moderate, and 25 studies were of weak quality. Among the quality rating criteria, 46 studies received a weak rating for selection bias because of their recruitment strategy or fewer than 60% of eligible participants taking part. Only 1 study received a strong rating for blinding, whereas the remaining studies either indicated blinding was not possible or did not report on blinding (Multimedia Appendix 2).

**Table 2:** Characteristics of studies.

Authors (year)	Study designs	Sample sizes	Outcome measures	Quality ratings
Agyapong et al (2017) [37]	Two-armed RCT^a^	73	BDI-II^b^	Strong
Anand et al (2016) [38]	Two-armed RCT	343	Myocardial Infarction Risk Score	Moderate
Arora et al (2014) [39]	Two-armed RCT	128	HbA_1c_^c^	Moderate
Bennett et al (2018) [40]	Two-armed RCT	351	Body weight	Weak
Bond et al (2010) [41]	Two-armed RCT	62	CES-D^d^ The Problem Areas in Diabetes Scale Diabetes Support Scale	Weak
Broekhuizen et al (2016) [42] and Wijsman et al (2013) [43]	Two-armed RCT	236	Research and Development 36-item health survey Ankle and wrist accelerometer	Moderate
Buller et al (2008) [44]	Two-armed RCT	755	Adapted all-day screener and self-report of servings	Moderate
Carroll et al (2019) [45]	Two-armed RCT	360	Patient Activation Measure	Weak
Caster et al (2017) [46]	One-group pretest-posttest	243	Knowledge scores	Weak
Chen et al (2016) [47]	Case study	1	Sleep satisfaction rating	Weak
Chen et al (2018) [48]	Two-armed RCT	233	Attendance rate Diabetic retinopathy Knowledge scores	Strong
Choi et al (2012) [49]^e^	Two-armed RCT	63	Chinese versions of Beck Depression Inventory Chinese bilingual version of PHQ-9^f^	Weak
Dang et al (2017) [50]	Two-armed (2:1) RCT	61	Self-Efficacy for Managing Chronic Disease	Moderate
Dear et al (2015) [51]^e^	Two-armed RCT	72	GAD-7^g^ PHQ-9	Weak
Dugas et al (2018) [52]	Five-armed RCT	27	HbA_1c_	Weak
Fortmann et al (2017) [53]	Two-armed RCT	126	HbA_1c_	Weak
Gilmore et al (2017) [54]	Two-armed RCT	40	Body weight	Moderate
Griffin et al (2018) [55]	One-group pretest-posttest	109	Body weight BMI	Weak
Hacking et al (2016) [56]	Two-armed RCT	223	Knowledge scores	Weak
Hageman et al (2014) [57]	Three-armed RCT	289	Blood pressure BMI Waist circumference	Moderate
Herring et al (2017) [58]	Two-armed RCT	66	Body weight	Moderate
Hill et al (2006) [59]^h^	Two-armed RCT	120	The Personal Resource Questionnaire Rosenberg Self-Esteem Scale Chronic Illness Empowerment Scale	Weak
Hong et al (2015) [60]	One-group pretest-posttest	30	Quality of life (self-reported seven-item questionnaire) Level of PA^i^	Weak
Ingersoll et al (2015) [61]	Two-armed RCT	63	Medication adherence (pharmacy refill data) Proportion of missed visits	Moderate
Jarvis et al (2019) [62]	Two-armed RCT	32	Disconnection and Rejection domains of the Young Schema Questionnaire de Jong Gierveld Loneliness Scale World Health Organization-Five Well-Being Index	Weak
Joseph et al (2015) [63]	Two-armed RCT	29	Sedentary behavior PA	Moderate
Kamal et al (2015) [64]	Two-armed RCT	200	Morisky Medication Adherence Scale	Moderate
King et al (2013) [65]	Two-armed RCT	40	Community Health Activities Model Program for Seniors questionnaire Daily steps	Moderate
Kiropoulos et al (2011) [66]	Two-armed RCT	202	Depression literacy scores Depression Stigma Scale BDI-II	Weak
Lee et al (2014) [67] and Lee et al (2016) [68]	One-group pretest-posttest	30	Adapted 15-item scale of Taylor et al [92] Intent (investigator developed questionnaire) Actual vaccination or test	Weak
Lee et al (2017) [69]	Two-armed RCT	131	Completed mammograms	Weak
MacDonell et al (2016) [70]	Two-armed RCT	49	Medication adherence Asthma control	Moderate
Marcus et al (2016) [71]	Two-armed RCT	205	Increased minutes/week of moderate to vigorous PA PA by accelerometer	Moderate
Mauriello et al (2016) [72]	Two-armed RCT	335	Self-reported behavior risks Daily fruit and vegetable consumption Daily minutes of stress management activity	Weak
Miller et al (2018) [73]	Two-armed RCT	450	Completed screening	Moderate
Moussa et al (2013) [74]	Two-armed RCT	45	Literacy Assessment for Diabetes	Moderate
Neafsey et al (2011) [75]	Two-armed RCT	160	The Adverse Self-Medication Behavior Risk Score	Moderate
Nelson et al (2016) [76]	One-group pretest-posttest	80	Diabetes Self-Care Activities Medication subscale	Weak
Neuenschwander et al (2013) [77]	Two-armed RCT	123	16-item questionnaire for low-income population for nutrition-related behavior outcomes	Moderate
Phelan et al (2017) [78]	Two-armed RCT	371	Body weight	Moderate
Rubinstein et al (2016) [79]	Two-armed RCT	637	Blood pressure	Moderate
Ryan et al (2013) [80]	One-group pretest-posttest	24	HbA_1c_ Cholesterol	Weak
Steinberg et al (2013) [81]	Two-armed RCT	50	Body weight	Moderate
Tessaro et al (2007) [82]	Two-armed RCT	395	34-item food frequency checklist Dietary knowledge	Moderate
Titov et al (2015) [83]^e^	Two-armed RCT	54	PHQ-9 GAD-7	Weak
Ünlü Ince et al (2013) [84]	Two-armed RCT	96	CES-D	Weak
Wahbeh et al (2016) [85]	Two-armed RCT	20	CES-D Five-Facet Mindfulness Questionnaire Positive and Negative Affect Schedule	Moderate
Wayne et al (2015) [86]	Two-armed RCT	97	HbA_1c_	Weak
Weinert et al (2008) [87]^h^	Three-armed RCT	176	Health knowledge score (investigator developed questionnaire)	Weak

^a^RCT: randomized controlled trial.

^b^BDI-II: Beck Depression Inventory II.

^c^HbA_1c_: hemoglobin A1c.

^d^CES-D: Center for Epidemiological Studies Depression.

^e^Adaptations of a similar intervention.

^f^PHQ-9: Patient Health Questionnaire nine-item.

^g^GAD-7: Generalized Anxiety Disorder seven-item scale.

^h^Same intervention but different cohorts.

^i^PA: physical activity.

### General Characteristics of the Interventions

Among the 48 interventions, 32 were from the United States, 4 from Australia, 3 from Canada, 2 from the Netherlands and South Africa, and 1 each from China, Malawi, Pakistan, and Taiwan, whereas 1 intervention was undertaken across 3 South American countries, namely, Argentina, Guatemala, and Peru. Low-income groups were the most common target group (n=20), followed by ethnic minorities (n=18), older adults (n=10), and rural communities (n=8). Low-literacy groups were targeted in 2 interventions [45,73]. A wide range of health issues were addressed among the 48 interventions, with diabetes being the most common (n=8), followed by 6 targeting physical inactivity and 5 targeting depression (Multimedia Appendix 3 [37-87]).

Websites were the most commonly used platforms, with 10 interventions using websites only and 12 interventions combining websites with other platforms such as email or text messaging. A total of 11 studies used text messaging alone, and 4 combined this with other platforms. A total of 10 interventions employed mobile apps. Facebook was used in 2 interventions, and WhatsApp was used in 1 intervention. Mobile phones were the most popular device, being used in 26 interventions, followed by the computer in 22 interventions. Tablets were used in 6 interventions.

Among the 48 interventions, 37 were interactive, providing information, tailored content, and/or health-engaging activities, and 11 were noninteractive, providing information or reminder text messages only. The duration of interventions ranged from one 30-min session to a 13-month program, with 3 months being the most common duration.

### Use of eHealth Literacy

No interventions explicitly reported that eHealth literacy needs were considered during the development, and no assessment of eHealth literacy was undertaken. In fact, eHealth literacy was only mentioned in a study by Carroll et al [45], which included eHealth literacy as one of the secondary outcome measures and used the eHealth Literacy Scale [93] for assessment. Apart from eHealth literacy, 4 interventions undertook other literacy assessments. Ingersoll et al assessed functional English literacy by using the Wide Range Achievement Test 4 [61], and health literacy was assessed in 3 interventions using different measures, including the Short Test of Functional Health Literacy in Adults [71], the Rapid Estimate of Adults Literacy in Medicine [75], or a single question [73]. All such assessments were conducted at baseline with no discussion as to whether baseline assessment played any role in intervention development (Table 3).

**Table 3:** The role of electronic health literacy and users in intervention development.

Authors (year)	Developmental frameworks	eHealth literacy or other literacy assessment or application of user-centered principles or user involvement
Agyapong et al (2017) [37]	Cognitive behavioral therapy principles	Content written in partnership with patients
Anand et al (2016) [38]	Integrative behavioral modification strategy Social cognitive social learning theories Goal setting theory Transtheoretical model	Pilot study
Arora et al (2014) [39]	Not reported	Not reported
Bennett et al (2018) [40]	Social cognitive theory Interactive obesity treatment approach	Not reported
Bond et al (2010) [41]	Not reported	Not reported
Broekhuizen et al (2016) [42] and Wijsman et al (2013) [43]	Transtheoretical model I-Change model	Not reported
Buller et al (2008) [44]	Social cognitive theory Diffusion of innovations model	Focus groups Usability testing
Carroll et al (2019) [45]	Capability, opportunity, motivation, and behavior model for behavior change Community-based participatory research	eHealth Literacy Scale used to measure eHealth literacy as one of the secondary outcomes Participatory research involving users
Caster et al (2017) [46]	Not reported	Focus groups
Chen et al (2016) [47]	Not reported	Not reported
Chen et al (2018) [48]	Not reported	Not reported
Choi et al (2012) [49]^a^	Adaptation of the sadness internet-delivered cognitive behavioral therapy program	Not reported
Dang et al (2017) [50]	Not reported	Not reported
Dear et al (2015) [51]^a^	Previous studies	Not reported
Dugas et al (2018) [52]	Not reported	Not reported
Fortmann et al (2017) [53]	Not reported	Not reported
Gilmore et al (2017) [54]	Learning theory Theory of planned behavior Theory of reasoned actions Social cognitive theory	Not reported
Griffin et al (2018) [55]	Social cognitive theory	Not reported
Hacking et al (2016) [56]	Social cognitive theory	Not reported
Hageman et al (2014) [57]	Pender’s Health Promotion Model based on social cognitive theory	Not reported
Herring et al (2017) [58]	Social cognitive theory Social ecological model	Focus groups Semistructured interviews
Hill et al (2006) [59] and Weinert et al (2008) [87]	Not reported	Pilot study
Hong et al (2015) [60]	Theory of goal setting	Usability testing
Ingersoll et al (2015) [61]	IMB^b^ model of adherence Social action theory	Functional English literacy assessed by Wide Range Achievement Test 4 Focus groups Interviews Usability testing
Jarvis et al (2019) [62]	Theoretical framework of loneliness Literature review Developed by a cognitive behavioral therapy specialist psychologist, a mental health nurse, and a mobile health expert	Not reported
Joseph et al (2015) [63]	Social cognitive theory	Not reported
Kamal et al (2015) [64]	The health belief model Social cognitive theory Michie’s taxonomy of behavioral change	Not reported
King et al (2013) [65]	Social cognitive theory Transtheoretical model	Participatory formative research
Kiropoulos et al (2011) [66]	Not reported	Not reported
Lee et al (2014) [67] and Lee et al (2016) [68]	Fogg behavioral model	Community advisory group Focus groups Usability testing
Lee et al (2017) [69]	Fogg behavioral model Health belief model Concept of persuasive technology	Community advisory group Focus groups Usability testing
MacDonell et al (2016) [70]	Principles of motivational interviewing IMB skills model	Pilot testing
Marcus et al (2016) [71]	Social cognitive theory Transtheoretical model	Health literacy assessed by the Short Test of Functional Health Literacy Focus groups
Mauriello et al (2016) [72]	Transtheoretical model of behavior change	Usability testing
Miller et al (2018) [73]	Not reported	Health literacy assessed by asking a single question, “how confident are you filling out medical forms by yourself?” Pilot testing
Moussa et al [74]	Not reported	Not reported
Neafsey et al (2011) [75]	Social cognitive theory	Health literacy assessed by Rapid Estimate of Adult Literacy in Medicine Usability testing Pilot testing
Nelson et al (2016) [76]	Adapted from the SuperEgo mobile communications platform	Usability testing
Neuenschwander et al (2013) [77]	Kolb’s learning styles and experiential learning model Use of the US Department of Health and Human Services’ Research-based Web Design and Usability Guidelines Previous users’ needs and requests	Pilot testing
Phelan et al (2017) [78]	Social cognitive theory Based on the diabetes prevention program and Look Ahead lifestyle interventions	Not reported
Rubinstein et al (2016) [79]	Transtheoretical model Health belief model	Focus groups Pilot study
Ryan et al (2013) [80]	Social cognitive theory	Not reported
Steinberg et al (2013) [81]	Interactive obesity treatment approach	Not reported
Tessaro et al (2007) [82]	Health belief model Social learning theory Social support theory	Focus groups
Titov et al (2015) [83]^a^	Psychological principles	Not reported
Ünlü Ince et al (2013) [84]	Not reported	Not reported
Wahbeh et al [85]	Modification of the mindfulness-based cognitive therapy and mindfulness-based stress reduction	Not reported
Wayne et al (2015) [86]	Motivational interviewing Cognitive behavioral therapy	Pilot study

^a^Adaptations of similar programs.

^b^IMB: Information, Motivation and Behavior Skills

### Use of Developmental Framework

Theoretical frameworks were the most used guidelines for developing interventions, with social cognitive theories (n=15) and the transtheoretical model (n=6) most commonly used. A total of 7 interventions were adaptations or modifications of previous programs, whereas 13 interventions did not provide any details about their theoretical frameworks or developmental frameworks. Only 1 intervention was reported on using the *Research-based Web Design and Usability Guidelines* developed by the US Department of Health and Human Services (UDHHS) [94] to inform the creation of their intervention website (Table 3) [77].

### User Involvement

User-centered principles were not discussed in any of the papers. The development of interventions in the included studies was mostly expert driven. A total of 22 interventions reported on involving users during development, with needs assessments using focus groups or interviews in 8 interventions, usability or pilot testing in 15 interventions, and 2 interventions mentioning participatory formative research with no details provided. Only Agyapong et al [37] reported that patients were involved in content writing (Table 3).

### Addressing eHealth Literacy Domains

Most reviewed interventions did not address all eHealth literacy domains. The number of domains addressed ranged from 1 to 7, with only 1 study containing components addressing all 7 eHealth literacy domains [65]. A total of 7 studies representing 6 interventions contained components of 6 domains [57,59,64,71,78,80,87], whereas 20 studies contained components addressing 5 domains (Multimedia Appendix 4 [37-87]).

**Figure 2:**
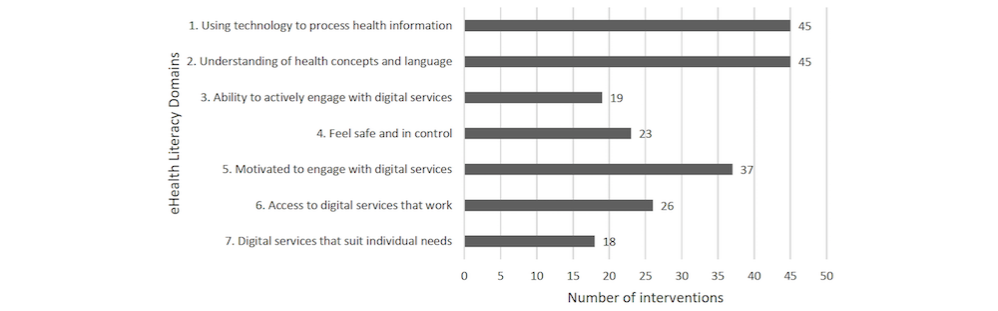
Number of interventions addressing the seven domains of the eHealth Literacy Framework [23, 25].

The 2 most addressed eHealth literacy domains, *1. Using technology to process health information* and *2.* *Understanding of health concepts and language*, were both identified in 45 interventions. The domain 5. *Motivated to engage with digital services* was addressed in 37 interventions through different strategies to encourage users to engage with interventions. A total of 26 interventions provided access to hardware, data plans, or technical support to address the domain *6. Access to digital services that work*, whereas 23 interventions supported the domain *4. Feel safe and in control* by requiring personal log-in or other forms of privacy measures. The 2 most overlooked domains were *3. Ability to actively engage with digital services* and *7. Digital services that suit individual needs*; both were identified in less than half of the 48 interventions. Of the 19 interventions containing components of *3. Ability to actively engage with digital services*, 15 provided training or instructions on using the intervention, whereas only 6 featured an easy-to-use navigation interface. Among the 18 programs addressing the domain *7. Digital services that suit individual needs*, the main strategy was to provide the preferred language of users. Accessibility features catering to individual capability or providing a user interface that suited individual needs such as large fonts or icons or audio options were only identified in 6 interventions [64,65,73-75,82]. Figure 2 shows the number of interventions addressing each of the 7 domains.

### Effectiveness of eHealth Interventions

Although no study explicitly considered or assessed eHealth literacy, the effectiveness of eHealth interventions was nevertheless examined, and the results were mixed. Approximately one-fourth of the reviewed interventions (n=13) did not find statistically significant improvements in their primary outcomes. Although 19 studies reported significant improvements in their primary outcomes, another 12 studies found significant improvements in some primary outcomes, but not all. In addition, 4 studies found improvements but did not report whether such differences were significant. The results of long-term effectiveness also produced mixed evidence. Among the 13 studies that conducted follow-up assessments, 8 found the effects sustainable up to a period of 12 months. A total of 3 studies found effects were not sustainable, whereas 2 did not report on the significance (Multimedia Appendix 3).

Among the reported effective 19 studies, there were no consistent patterns of intervention characteristics or eHealth literacy domains likely addressed. These interventions could be interactive or noninteractive, although platforms and devices also varied. The number of eHealth literacy domains likely addressed ranged from 1 to 7. Although a study by King et al [65] likely addressed all 7 domains and found their intervention effective with a large effect size (0.8-1.2), a study by Chen et al [48] also reported their study as effective, although only the domain *1. Using technology to process health information* could be identified within the intervention components.

## Discussion

### Principal Findings

Although the concept of eHealth literacy was introduced more than a decade ago [17], this review finds that utilization of the concept for enhancing eHealth use and engagement is rarely recognized. The eHealth literacy needs of users were not explicitly considered during intervention development in any of the included studies, and no eHealth literacy assessment was conducted to ensure that such needs were met. This result is echoed in an earlier systematic review of eHealth and telehealth tools for vulnerable populations, which reported that eHealth literacy was not assessed in any of the 18 included studies [95]. In fact, eHealth literacy is only mentioned in 1 of the 51 papers of this review. Although 3 studies conducted health literacy assessments at baseline [71,73,75] and 1 assessed eHealth literacy as a secondary outcome [45], the results were not used for intervention development. The fact that eHealth literacy is overlooked may be because of the lack of comprehensive measures before 2018 and an associated knowledge gap in using such assessment to inform eHealth design [96]. To move forward, in-depth research on eHealth literacy is required, such as the application of the eHLF and the recently developed comprehensive eHLQ, designed to support eHealth intervention development and evaluation. Developed on the basis of eHLF, the eHLQ is a 35-item questionnaire that produces 7 scores representing 7 eHealth literacy domains of users. The resulting scores provide insights into users’ strengths and weaknesses in using eHealth such that interventions can be tailored accordingly. For example, if target users reported good ability to use technology (higher scores in *3. Ability to actively engage with digital services*) but lack motivation (lower scores in *5. Motivated to engage with digital services*), features to address motivation should be a prominent feature of the intervention. However, if target users demonstrated limited ability to use technologies, interventions such as simple unidirectional text messages, rather than interactive mobile apps, are likely to be more suitable for the target users. Hence, the eHLF and eHLQ will have the potential to advance the field of eHealth literacy and strengthen the reach and impact of digital health interventions [25].

### Addressing eHealth Literacy Needs

There is growing concern that frameworks or guidelines informing the development of eHealth interventions so that they meet users’ needs are lacking [97], and this concern is reflected in the findings of this review. Only 1 study [77] used the *Research-based Web Design and Usability Guidelines* by the UDHHS to inform intervention development. However, the guideline authors specifically indicate that they may not be applicable to all audiences, such as people with low literacy who may have different reading and layout needs [94]. In addition, only 22 studies in this review discussed user involvement whereas needs assessments were usually in the form of focus groups or interviews involving a limited number of users. Only 1 study reported the inclusion of patients in content writing [37]. Such practice means that interventions are expert driven instead of user driven, echoing the concern that users and patients are the most underused resources in developing eHealth interventions [98]. Although eHealth literacy is only one of the factors in developing effective eHealth strategies, it has been advocated that it is a primary and critical factor that affects usability and adoption [17,21,26]. Even if an intervention is grounded in theory, it will not be usable if it does not align with the literacy needs and abilities of end users and may lead to nonadoption [30,99,100]. Hence, research efforts into eHealth developmental frameworks incorporating eHealth literacy need assessment, and user-centered principles are required such that equal access and usage can be achieved for all users.

Although eHealth literacy needs may not be explicitly considered when developing eHealth programs, this review still finds that interventions generally have features that may meet eHealth literacy needs based on the eHLF. However, the common focus is on providing information or features that address the domains of *1. Using technology to process health information*, *2. Understanding of health concepts and language*, and *5. Motivated to engage with digital services*. Strategies to assist users in using or engaging with technology and accessibility features of systems that are tailored and responsive to an individual’s ability and capability are generally overlooked. These findings resonate with those from a systematic review of diabetes apps targeted at older adults that there is a limited variety of accessibility features [101]. This is of special importance when an intervention is designed for older people or people with disabilities who may require specialized tools because of functional and cognitive impairments [102,103] or people with low literacy skills who may have different reading and design needs [94]. In addition, applying the eHLF to determine whether certain eHealth literacy domains were addressed may not necessarily mean that the eHealth literacy needs of users were met as the actual eHealth literacy needs of target users were not assessed and, therefore, not known. The results highlight that in developing interventions using technologies, designers are mainly responsible for ensuring that users’ needs and capabilities are met in the hope that users will adopt the intervention to improve or change their health behavior. However, Chang et al [104] noted that eHealth intervention designers were typically not trained to meet the communication needs of underserved communities. Showell et al [105] also pointed out that eHealth systems tended to be designed for users who were similar to the designers, who were usually middle-class professionals. As such, the needs of disadvantaged patients were generally overlooked in the design process [15].

### Effectiveness of eHealth Interventions

In addition to exploring the role of eHealth literacy and eHealth intervention development, this review also examined the effectiveness of eHealth interventions targeted at socially disadvantaged groups and found inconclusive evidence. Although significant improvements were found in 19 studies, these findings should be interpreted with caution, as 10 studies are of weak quality and 7 studies are of moderate quality. Although 3 studies reported a large effect size, they had smaller sample sizes and were of moderate or weak quality [51,65,83]. The sustainability of effects is also mixed and cannot be ascertained, as most studies have short follow-up times. These findings are similar to reviews of eHealth interventions, which also report inconclusive evidence on effectiveness [35,103,106,107]. The lack of comprehensive eHealth literacy assessments also prevents this review from exploring the link between eHealth literacy and the effectiveness of eHealth programs. Further robust empirical studies need to be undertaken to better understand the role of eHealth literacy in eHealth interventions to help address the digital divide and improve health disparities.

### Limitations

Several limitations of this review need to be acknowledged. Only peer-reviewed journals were included for this review, and there may be other studies that were not accounted for. The search was conducted by one researcher, which may have led to potential bias. The findings of this review may not reflect all details of the actual intervention, as authors generally only briefly describe their intervention development processes [108], and few studies report how users are involved such that interventions are aligned with their needs [27]. However, not reporting certain features suggests that the authors may not consider such features as relevant. Furthermore, the included studies do not represent all socially disadvantaged groups. This review only focused on certain categories of disadvantaged groups and did not include other underserved populations, such as people with disabilities or indigenous people who may also have limited access or skills to use ICT [109]. Future reviews should consider inclusion of these groups to advance eHealth research among vulnerable populations.

### Conclusions

The WHO recognizes health literacy as a critical determinant of health that has the potential to empower individuals and bring about health equity [19]. However, this systematic review finds that the role of eHealth literacy in designing eHealth interventions targeted at socially disadvantaged groups is generally overlooked. eHealth literacy was not explicitly considered or assessed during intervention development. There was also a lack of frameworks or theories informing eHealth designers on how to meet users’ needs. Although users were involved in some of the reviewed studies, intervention development was mainly expert driven rather than user driven. By using the eHLF to examine the eHealth literacy components of eHealth interventions, it was found that the design of features such that they suited individual capability was not common. Furthermore, whether the eHealth literacy needs of users were actually addressed in the reviewed interventions cannot be ascertained because of the lack of comprehensive eHealth literacy assessment. The link between eHealth literacy and effectiveness of eHealth interventions cannot be explored. Moreover, the paucity of robust studies also delivers limited empirical evidence on how to effectively reach these vulnerable populations and bridge the digital divide.

Despite the concept of eHealth literacy being introduced in 2006, its potential role in empowering individuals has not been realized. Without meeting the eHealth literacy needs of disadvantaged groups, adoption of eHealth interventions is likely to be low, resulting in ineffective interventions [17,21,30,99,100,110]. To ensure that no one is left behind as determined in the Shanghai Declaration on Promoting Health [19], eHealth literacy must be acknowledged and included in the development of eHealth interventions to assist the realization of technological advancement and improve health equity.

## Appendix

Multimedia Appendix 1 Search strategies.

Multimedia Appendix 2 Quality assessment.

Multimedia Appendix 3 Intervention characteristics and key findings.

Multimedia Appendix 4 Summary of eHealth literacy domains likely addressed.

## Abbreviations

eHLF: eHealth Literacy Framework
eHLQ: eHealth Literacy Questionnaire
ICT: information and communications technology
RCT: randomized controlled trial
UDHHS: US Department of Health and Human Services
WHO: World Health Organization
