# Supplementary material for: Applying the Electronic Health Literacy Lens: Systematic Review of Electronic Health Interventions Targeted at Socially Disadvantaged Groups
Source: J Med Internet Res. 2020 Aug 13;22(8):e18476. doi: 10.2196/18476 (PMC7453328; doi:10.2196/18476)
Supplement: Multimedia Appendix 1 [file jmir_v22i8e18476_app1.docx]

Multimedia Appendix 1: Search strategies

| Number | Search term |
| --- | --- |
| Academic Search Complete, AgeLine, CINAHL Complete, Communication & Mass Media Complete, ERIC, Global Health, MEDLINE Complete, PsycINFO and SocINDEX via EBSCOhost | |
| 1 | eHealth OR e-Health OR mHealth OR “digital health” |
| 2 | health N3 (“web based” OR “internet based” OR online OR internet OR “cell phone*” OR “mobile phone*” OR smartphone* OR “smart phone*” OR “computer based”) |
| 3 | 1 OR 2 |
| 4 | intervention* OR program* OR application* OR app OR apps |
| 5 | “older adult*” OR elder* OR senior* OR aged OR “older people”  Narrow by Age: very old (85 yrs & older), aged, 80 & over, aged (65 yrs & older), middle age (40 – 64 yrs), 65+ years, middle aged: 45 – 64 years |
| 6 | “low literacy” OR “low education” |
| 7 | Migrant* OR Immigrant* OR refugee* OR “minority population*” OR “racial minorit*” OR “ethnic minorit*” OR “culturally and linguistically diverse” |
| 8 | rural OR regional OR “remote area*” |
| 9 | “low income” OR “limited income” OR "low socioeconomic" OR poor OR “socioeconomic disadvantage*” |
| 10 | “socially disadvantaged” OR disadvantaged OR vulnerable OR underserved OR “underserved person*” |
| 11 | 3 AND 4 AND 5 |
| 12 | 3 AND 4 AND 6 |
| 13 | 3 AND 4 AND 7 |
| 14 | 3 AND 4 AND 8 |
| 15 | 3 AND 4 AND 9 |
| 16 | 3 AND 4 AND 10 |
| EMBASE | |
| 1 | eHealth OR e-Health OR mHealth OR ‘digital health’ |
| 2 | health NEAR/3 (‘web based’ OR ‘internet based’ OR online OR internet OR ‘cell phone*’ OR ‘mobile phone*’ OR smartphone* OR ‘smart phone*’ OR ‘computer based’) |
| 3 | 1 OR 2 |
| 4 | intervention* OR program* OR application* OR app OR apps |
| 5 | ‘older adult*’ OR elder* OR senior* OR aged OR ‘older people’  Limit by: middle aged OR aged OR very elderly |
| 6 | ‘low literacy’ OR ‘low education’ |
| 7 | Migrant* OR Immigrant* OR refugee* OR ‘minority population*’ OR ‘racial minorit*’ OR ‘ethnic minorit*’ OR ‘culturally and linguistically diverse’ |
| 8 | rural OR regional OR ‘remote area*’ |
| 9 | ‘low income’ OR ‘limited income’ OR ‘low socioeconomic’ OR poor OR ‘socioeconomic disadvantage*’ |
| 10 | ‘socially disadvantaged’ OR disadvantaged OR vulnerable OR underserved OR ‘medically underserved person*’ |
| 11 | 3 AND 4 AND 5 |
| 12 | 3 AND 4 AND 6 |
| 13 | 3 AND 4 AND 7 |
| 14 | 3 AND 4 AND 8 |
| 15 | 3 AND 4 AND 9 |
| 16 | 3 AND 4 AND 10 |
