# Supplementary material for: Applying the Electronic Health Literacy Lens: Systematic Review of Electronic Health Interventions Targeted at Socially Disadvantaged Groups
Source: J Med Internet Res. 2020 Aug 13;22(8):e18476. doi: 10.2196/18476 (PMC7453328; doi:10.2196/18476)
Supplement: Multimedia Appendix 2 [file jmir_v22i8e18476_app2.docx]

**Multimedia Appendix 2: Quality assessment**

| Authors (Year) | Selection Bias | Study Design | Confounders | Blinding | Data Collection Methods | Withdrawals and Drop-outs | Global Rating |
| --- | --- | --- | --- | --- | --- | --- | --- |
| Agyapong et al (2017) [37] | Moderate | Strong | Strong | Moderate | Strong | Strong | Strong |
| Anand et al (2016) [38] | Weak | Strong | Strong | Moderate | Strong | Strong | Moderate |
| Arora et al (2014) [39] | Weak | Strong | Strong | Moderate | Strong | Moderate | Moderate |
| Bennett et al (2018) [40] | Weak | Strong | Strong | Weak | Strong | Strong | Weak |
| Bond et al (2010) [41] | Weak | Strong | Strong | Moderate | Strong | Weak | Weak |
| Broekhuizen et al (2016) [42] | Weak | Strong | Strong | Moderate | Strong | Strong | Moderate |
| Buller et al (2008) [44] | Weak | Strong | Strong | Moderate | Strong | Moderate | Moderate |
| Carroll et al (2019) [45] | Weak | Strong | Strong | Weak | Strong | Strong | Weak |
| Caster et al (2017) [46] | Moderate | Moderate | N/A | Weak | Weak | Weak | Weak |
| Chen et al (2016) [47] | N/A | Weak | N/A | N/A | Weak | N/A | Weak |
| Chen et al (2018) [48] | Moderate | Strong | Strong | Moderate | Strong | Strong | Strong |
| Choi et al (2012) [49] | Weak | Strong | Strong | Weak | Strong | Strong | Weak |
| Dang et al (2017) [50] | Weak | Strong | Strong | Moderate | Strong | Strong | Moderate |
| Dear et al (2015) [51] | Weak | Strong | Strong | Weak | Strong | Strong | Weak |
| Dugas et al (2018) [52] | Weak | Strong | Weak | Weak | Strong | Strong | Weak |
| Fortmann et al (2017) [53] | Weak | Strong | Strong | Weak | Strong | Strong | Weak |
| Gilmore et al (2017) [54] | Weak | Strong | Strong | Moderate | Strong | Strong | Moderate |
| Griffin et al (2018) [55] | Weak | Moderate | N/A | Weak | Strong | Moderate | Weak |
| Hacking et al (2016) [56] | Weak | Strong | Strong | Moderate | Weak | Moderate | Weak |
| Hageman et al (2014) [57] | Weak | Strong | Strong | Moderate | Strong | Strong | Moderate |
| Herring et al (2017) [58] | Weak | Strong | Strong | Moderate | Strong | Strong | Moderate |
| Hill et al (2006) [59] | Weak | Strong | Weak | Moderate | Strong | Strong | Weak |
| Hong et al (2015) [60] | Weak | Moderate | N/A | Weak | Weak | Strong | Weak |
| Ingersoll et al (2015) [61] | Weak | Strong | Strong | Moderate | Strong | Moderate | Moderate |
| Jarvis et al (2019) [62] | Weak | Strong | Strong | Weak | Strong | Strong | Weak |
| Joseph et al (2015) [63] | Weak | Strong | Strong | Moderate | Strong | Strong | Moderate |
| Kamal et al (2015) [64] | Weak | Strong | Strong | Moderate | Strong | Strong | Moderate |
| King et al (2013) [65] | Weak | Strong | Strong | Moderate | Strong | Strong | Moderate |
| Kiropoulos et al (2011) [66] | Weak | Strong | Strong | Weak | Strong | Strong | Weak |
| Lee et al (2014) [67] | Weak | Moderate | N/A | Weak | Weak | Strong | Weak |
| Lee et al (2016) [68] | Weak | Moderate | N/A | Weak | Weak | Strong | Weak |
| Lee et al (2017) [69] | Weak | Strong | Strong | Weak | Strong | Strong | Weak |
| MacDonnell et al (2016) [70] | Weak | Strong | Strong | Moderate | Strong | Strong | Moderate |
| Marcus et al (2016) [71] | Weak | Strong | Strong | Moderate | Strong | Strong | Moderate |
| Mauriello et al (2016) [72] | Moderate | Strong | Strong | Weak | Weak | Moderate | Weak |
| Miller et al (2018) [73] | Weak | Strong | Strong | Strong | Strong | Strong | Moderate |
| Moussa et al (2013) [74] | Weak | Strong | Strong | Moderate | Strong | Strong | Moderate |
| Neafsey et al (2011) [75] | Weak | Strong | Strong | Moderate | Strong | Strong | Moderate |
| Nelson et al (2016) [76] | Weak | Moderate | N/A | Weak | Strong | Weak | Weak |
| Neuenschwander et al (2013) [77] | Weak | Strong | Strong | Moderate | Strong | Strong | Moderate |
| Phelan et al (2017) [78] | Weak | Strong | Strong | Moderate | Strong | Strong | Moderate |
| Rubinstein et al (2016) [79] | Weak | Strong | Strong | Moderate | Strong | Strong | Moderate |
| Ryan et al (2013) [80] | Weak | Moderate | N/A | Weak | Strong | Weak | Weak |
| Steinberg et al (2013) [81] | Weak | Strong | Strong | Moderate | Strong | Strong | Moderate |
| Tessaro et al (2007) [82] | Weak | Strong | Strong | Moderate | Moderate | Moderate | Moderate |
| Titov et al (2015) [83] | Weak | Strong | Strong | Weak | Strong | Moderate | Weak |
| Ünlü Ince et al (2013) [84] | Weak | Strong | Strong | Moderate | Strong | Weak | Weak |
| Wahbeh et al (2016) [85] | Weak | Strong | Strong | Moderate | Strong | Moderate | Moderate |
| Wayne et al (2015) [86] | Weak | Strong | Strong | Weak | Strong | Moderate | Weak |
| Weinert et al (2008) [87] | Weak | Strong | Strong | Moderate | Weak | Weak | Weak |
| Wijsman et al (2013) [43] | Weak | Strong | Strong | Moderate | Strong | Strong | Moderate |
