# Supplementary material for: Applying the Electronic Health Literacy Lens: Systematic Review of Electronic Health Interventions Targeted at Socially Disadvantaged Groups
Source: J Med Internet Res. 2020 Aug 13;22(8):e18476. doi: 10.2196/18476 (PMC7453328; doi:10.2196/18476)
Supplement: Multimedia Appendix 3 [file jmir_v22i8e18476_app3.docx]

**Multimedia Appendix 3: Intervention characteristics and key findings**

| Authors (Year) & Country | Target Group(s) & Health issue | Platform & Device | Intervention | Key Findings |
| --- | --- | --- | --- | --- |
| Agyapong et al (2017) [37]  Canada | Rural communities  Depression | Text message  Mobile phone | Supportive text messages for depression  IG: Received messages formulated to target mood improvement, twice daily unidirectional with no repetition of messages, same messages for all participants  CG: No intervention | - IG showed significantly lower depression scores than CG (20.8 (SD = 11.7) vs. 24.9 (SD 11.5), *F* (1, 60) = 4.83, *P* = .03, np2 = .07) with an effect size (Cohen’s *d*) of .67 |
| Anand et al (2016) [38]  Canada | Ethnic minorities (South Asian Canadian)  Myocardial Infarction (MI) | Website & email & text messages  Computer & mobile phone | SAHARA (South Asian HeArt Risk Assessment) Trial  IG: Received bi-weekly email health messages using a strategy of goal setting and continual re-enforcement based on stages of change that support motivation and confidence for behavior change and self-monitoring, targeted at dietary intake and physical activity, health tips via email or text messages weekly, encouraged to use the SAHARA website to access South Asian-specific prevention advice  CG: Usual care | - No significant difference in Myocardial Infarction risk score between IG and CG (.27, 95% *CI*: -1.12 to 0.58, *P* = .53) |
| Arora et al (2014) [39]  USA | Low-income groups & Ethnic minorities (Latino American)  Diabetes | Text message  Mobile phone | TExT-MED (Text Message for ED Patients with Diabetes)  IG: 2 daily text messages from 4 categories: educational/ motivational (1 per day), medication reminders (3 per week), healthy living challenges (2 per week), and trivia (2 per week) with answers sent out 1 hour after the trivia message, messages also available in Spanish  CG: Usual care | - No significant difference in HbA1c between IG and CG (*P* = .23) |
| Bennett et al (2018) [40]  USA | Low-income groups  Obesity | Mobile app  Mobile phone | Track  IG: Program involved six components: 1) tailored behavioral goals; 2) used the Track app to self-monitor such goals; 3) used a cellular-connected scale for self-weighing; 4) skills training information; 5) 18 weight loss counselling calls; and 6) brief healthcare provider-delivered weight loss counselling  CG: Usual care | - IG showed significantly greater mean weight change than CG (mean difference: -3.8 kg, 95% *CI*: -5.1 to -2.5 kg, *P* < .001) |
| Bond et al (2010) [41]  USA | Older adults  Diabetes and Psychosocial wellbeing | Website & email  Computer | Web-based psychosocial well-being intervention  IG: Website where participants could enter a log of self-management activities to be reviewed by study nurse, who would then provide tailored self-management feedback, provision of information on diabetes management, diet, exercise and ways to deal with physical and emotional demands of diabetes, participants could join a weekly online discussion group facilitated by study nurse for peer support to share experience or ask questions, also had access to one another through email and instant message  CG: Usual care | - Univariate ANCOVA F tests also show a significant difference for depression and quality of life (*P* < .05) as well as social support (*P* < .001) with effect size (Cohen’s *d*) of .7 for depression and quality of life and 1.0 for social support |
| Broekhuizen et al (2016) [42]  Wijsman et al (2013) [43] | The Netherlands  Older adults  Physical Inactivity | Website  Computer | DirectLife  IG: Program consisted of three components: 1) activity monitor uploaded daily; 2) a personal website; and 3) a personal eCoach giving advice on how to increase PA and answered questions, personalized targets set by program but participants could vary the goals  CG: Waitlist | - No significant difference between IG and CG on overall Quality of Life scores (*P* = .52) - Ankle and wrist accelerometer recorded IG had significantly more increase in moderate-to-vigorous activity than CG (*P* = .001) |
| Buller et al (2008) [44]  USA | Rural communities  Nutrition | Website & email  Computer | 5 a Day, the Rio Grande Way Website  IG: Nutrition information with practical skills and resources presented in various formats  CG: Waitlist | - No significant difference in fruits and vegetable intake between IG and CG (estimated difference = 0.64, *SD* = 0.52, *t*(*df* = 416) = 1.22, *P* = .223) |
| Carroll et al (2019) [45]  USA | Low-income groups & Low-literacy groups & Ethnic minorities  HIV | Mobile app  iPod | Get Ready and Empowered About Treatment (GREAT)  IG: Provision of customized electronic Personal Health Records (ePHR) called URHealth using Apple iPod, 6 x 90-minute peer-led group training sessions on how to use the iPod, ePHR and search for online health information and a final coaching session taught by a peer trainer  CG: Usual care | - IG showed significantly greater improvement in patient activation than CG in (difference 2.82, 95% *CI* .32 - 5.32, *P* = .027) at 12 months |
| Caster et al (2017) [46]  Malawi | Rural communities  Cervical cancer | Mobile app  Tablet | Educational intervention on cervical cancer  Intervention: Content of the program included: 1) anatomical and etiological information; 2) information on local availability of screening and treatment; and 3) correction of misconceptions | - Significant increase in median knowledge scores from 11 to 18 (*P* < .001) |
| Chen et al (2016) [47]  Taiwan | Older adults  Insomnia | Mobile app  Tablet | Win-Win aSleep (WWaS)  Intervention: Consisted of 4 modules: 1) the 'reminding module' to adhere to prescribed interventions and activities; 2) the 'guiding module' to provide relaxation multimedia to guide practice; 3) the 'e-Diary module' as a digital sleep diary; and 4) the 'visualization' module to provide a summary of sleep information | - Sleep satisfaction rating (0 = very bad to 3 = very good) increased from 1.5 (*SD* = .7) at baseline to 2.0 (*SD* = .0) at post-intervention (significance not reported) |
| Chen et al (2018) [48]  China | Low-income groups & Rural communities  Diabetic retinopathy (DR) | Text message  Mobile phone | SMS (Short Message Service) information reminders for eye examination  IG: At least 3 SMS appointment reminders at 1 week and 3 days before the scheduled appointment, combined with information on diabetic retinopathy and the need for eye examination  CG: No intervention | - IG showed significantly higher attendance rate than CG (mean difference: 28.8%, 95% *CI*: 17.9% to 39.8%, *P* < .001) - IG showed significantly higher diabetic retinopathy knowledge scores than CG (mean difference 1.30, 95% *CI*: .96 to 1.63, *P* < .001) |
| Choi et al (2012) [49]^a^  Australia | Ethnic Minorities (Chinese Australian)  Depression | Website & email  Computer | Brighten Your Mood Program  IG: Received 6 educational lessons of best practice principles of CBT programs with homework assignment for each lesson, additional written resources relating to depression, weekly telephone contact and email with support personnel, information also available in Chinese  CG: Waitlist | - At post-intervention:   IG showed significantly lower Depression scores (*F*_1, 52_ = 30.69, *P* < .001) with effect size (Cohen’s *d*) of .93 (−4.57 to 1.88) and CB-PHQ-9 scores (*F*_1, 52_  = 5.28, *P* = .026) with effect size (Cohen’s *d*) of .50 (−2.37 to .81) than CG   - At 3-month follow-up:   IG showed no change in CBDI score between post-treatment and follow-up (*t*_24_ range = -2.07 - 5.91, *P* > .05) and a significant reduction in CB-PHQ-9 scores (*t*_24_ range = .14 - 4.42, *P* = .038) (CG joined program and not assessed) |
| Dang et al (2017) [50]  USA | Ethnic minorities (African and Latino Americans)  Chronic heart failure | Text message  Mobile phone | Heart Failure monitoring text messaging  IG: 10 daily questions sent through an automated system using simple yes-no responses to monitor signs and symptoms of heart failure  CG: Usual care | - IG showed significantly higher self-efficacy scores than CG (2.09 ± 2.32, *P* = .005) |
| Dear et al (2015) [51]^a^  Australia | Older adults  Anxiety | Website & email  Computer | Managing Stress and Anxiety Course  IG: Received 5 lessons to learn and practice psychological skills, homework assignment for each lesson, additional resources relating to communication and skills, regular reminder and email messaging from therapist  CG: Waitlist | - At post-intervention:   IG showed significantly lower scores on anxiety (Cohen's *d* = 1.43; 95% *CI*: .89-1.93, *P* < .001) and depression (Cohen's *d* = 1.79; 95% *CI*: 1.21 to 2.32, *P* < .001) than CG   - At 3-month and 12-month follow-up:   IG showed no significant change in anxiety and depression scores (*P* s > .05) |
| Dugas et al (2018) [52]  USA | Older adults  Diabetes | Mobile app  Mobile phone | DiaSocial  IG: Provision of specific and salient health behavior goals through gamification point system and self-monitoring of health behavior. IG1 received app only intervention, IG2 received app with clinician engagement, IG3 received app with peer engagement and IG4 received app with clinician and peer engagement  CG: Usual care | - No significant differences in HbA1c between all groups |
| Fortmann et al (2017) [53]  USA | Ethnic minorities (Latino American) & Low-income groups  Diabetes | Text message  Mobile phone | Dulce Digital (SMS for glycemic)  IG: Received culturally appropriate educational materials and motivational messages, prompts for medication, and blood glucose monitoring  CG: Usual care | - IG showed a significantly lower mean HbA1c level than CG (8.5 ± 1.2% [69.0 ± 13.1 mmol/mol] vs 9.4 ± 2.0% [78.0 ± 20.8 mmol/mol], *P* = .03) |
| Gilmore et al (2017) [54]  USA | Low-income groups  Postpartum weight retention | Mobile app  Mobile phone | E-Moms – Smartloss  IG: A personalized lifestyle intervention with near real-time monitoring of weight and activity, scheduled delivery of health information and feedback, quick receipt of information about adherence to prescribed diet and/or exercise goal, and synchronous communication with a health care professional  CG: Usual care | - No significant weight difference (*P* = .10) between IG (1.8 ± 0.9 kg, *P* = .05) and CG (-0.1 ± 0.9 kg, *P* = .92) |
| Griffin et al (2018) [55]  USA | Low-income groups  Diet and physical inactivity | Text message  Mobile phone | My Quest  Intervention: Received text messages and eNewsletters with information on diet and physical activity, goal setting, and self-monitoring of weight and physical activity | - Significant reduction in mean body weight (204.3 vs 199.6 lb; *t*[55] = 3.047; *P* = .004) at post-intervention - Significant reduction in mean BMI (34.7 vs 33.9; *t*[55] = 3.254; *P* = .002) at post-intervention |
| Hacking et al (2016) [56]  South Africa | Low-income groups  Hypertension | Text message  Mobile phone | SMS on hypertension information  IG: Text messages with health information and tips on diet and exercise  CG: Usual care | - No significant difference in knowledge between groups (*P* = .69) |
| Hageman et al (2014) [57]  USA | Rural communities  Pre-hypertension | Website  Computer | Wellness for Women: DASHing towards Health  IG1: Participants could self-record blood pressure, eating and activity on website, 18 newsletters with tailored messages, self-assessment quizzes and training videos, also supported with 2-hour face-to-face training sessions by dietitian, telephone counselling for goal-setting  IG2: Received print-based intervention  CG: Standard advice | - At post-intervention:   No significant differences between groups across blood pressure, BMI and waist circumference   - At 12-month follow-up:   No significant differences between groups across blood pressure, BMI and waist circumference |
| Herring et al (2017) [58]  USA | Ethnic minorities (African American) & Low-income groups  Postpartum weight retention | Facebook & Text message  Computer & mobile phone | Technology-based behavior intervention for weight control  IG: Using empirically supported behavior change goals to target self-weighing, physical activity and energy intake, interactive self-monitoring text messages, skills training and support via Facebook, also supported by weekly to monthly health coach calls  CG: Usual care | - At post-intervention:   IG participants were significantly more likely to be at or below their early pregnancy weights compared to CG (56% vs. 29 %, *P* = .04)   - At 6-month follow-up:   No significant difference between proportion of women in IG and CG (41% vs. 38%, *P* = .83) at (within .9 kg) or below early pregnancy weights |
| Hill et al (2006) [59]  Weinert et al (2008) [87]  USA | Rural communities  Chronic health conditions | Website & email  Computer | Women to Women project  IG of 2006 study: An online self-help support group developed to foster social support and teach participants the computer literacy skills required to find and evaluate online health information, also included an email function, chat room function, and additional resources  IG1 of 2008 study: same intervention as 2006 study but with additional expert-facilitated discussions  IG2 of 2008 study: same intervention as 2006 study  CG for both 2006 and 2008 studies: No intervention | - 2006: IG showed significantly higher scores than CG for social support, *F* (1,98) = 4.43, *P* = .038, self-esteem, *F* (1.98) = 5.97, *P* = .016, and empowerment, *F* (1,98) = 6.06, *P* = .016 - 2008: IG1 had significantly higher health knowledge acquisition than CG (Mean difference = .107, *P* = .000) and IG2 also had significantly higher knowledge acquisition than CG (Mean difference = .112, *P* = .000) but no significant difference between IG1 and IG2 (Mean difference = -.005, *P* = .963). Mean knowledge scores of IG1 and IG2 dropped after 6 months |
| Hong et al (2015) [60]  USA | Older adults  Physical inactivity | Mobile app  Mobile device or computer | iCanFit  Intervention: Major functions included ‘goals’, ‘community’, ‘tips’ and ‘resources’, participants were motivated to regular exercise through goals setting, activity tracking, personalized feedback, and progress reviews, could enter activity and record the total number of minutes on an interactive calendar, tailored messages based on the progress in meeting goals, secure online network to connect with other cancer survivors | - Significant difference in overall quality of life score (*P* = .004) at post-intervention - No significant difference in level of physical activity at post-intervention |
| Ingersoll et al (2015) [61]  USA | Rural communities  HIV | Text message  Mobile phone | TEXT (Treatment Extension by TEXT)  IG: Received bidirectional text messaging with tailored responses based on replies, daily queries of medication dosing, mood (twice) and substance use (once)  CG: Usual care | - At post-intervention:   IG showed significantly better ART adherence than CG (P = .04)  No significant difference between groups in proportion of missed visits (P = .12)   - At 3-month follow-up:   No significant difference between groups for both measures |
| Jarvis et al (2019) [62]  South Africa | Older adults  Maladaptive cognitions | WhatsApp  Mobile phone | Living In Network Connected Communities (mLINCC)  IG: Involved 4 phases. 1) two weeks: selection of a smartphone and 4 x 90-minute face-to-face group training sessions, 2) two weeks: involved 4 x 90-minute face-to-face sessions on loneliness and key messages were confirmed through WhatsApp messages, 3) 1 month: individualized WhatsApp messages with positively framed content about maladaptive cognitions, 4) 1 month: weekly Help Desk but no more messages  CG: Usual care | - No significant difference between IG and CG in total social cognition score, total loneliness score and mental well-being score at baseline and post-intervention - Significant difference between IG and CG at baseline and one month follow-up in total social cognition score (IG: -29.54±25.18 vs CG: 4.18±29.13, *P* = .006, effect size, Hedge’s g = 1.23) and total loneliness score (IG: -2.08 ± 1.75 vs CG: 0.41 ± 1.58, *P* < .001, effect size, Hedge’s g = 1.50) but not mental well-being score |
| Joseph et al (2015) [63]  USA | Ethnic minorities (African American)  Physical inactivity | Facebook, email & text message  Mobile phone | Facebook, email and text message program  IG: Physical activity promotion materials posted on Facebook wall every week, also posted discussion topics and encourage participant engagement, motivational mobile phone text messages to promote physical activity, adaptive self-monitoring using pedometer and goal-setting, weekly individualized step goals and social reinforcement sent through email  CG: Standard advice | - IG showed significantly decreased sedentary behavior (*P* = .026), increased light-intensity PA (*P* = .024) and moderate-lifestyle intensity PA (*P* < .001) than CG, measured by accelerometer - No significant differences between groups in moderate-intensity PA (*P* = .422), moderate-to-vigorous PA (*P* = .317) nor moderate-to-vigorous intensity PA in bouts of 10-minutes or greater (*P* = .637) |
| Kamal et al (2015) [64]  Pakistan | Low-income groups  Stroke | Text message  Mobile phone | SMS4Stroke  IG: Received automated text messages reminders customized to the participants' medication, needed to respond whether medication was taken or not, dissemination of at least two weekly health information text messages  CG: Usual care | - IG showed significantly higher medication adherence scores than CG (.54, 95% *CI*: .22 to .85, *P* < .01) |
| King et al (2013) [65]  USA | Ethnic minorities & Low-income groups  Physical inactivity | Website  Computer | ECA (Embodied Conversational Agent)  IG: Participants could use the program anytime when they visited the center during the study period, each session involved individualized and culturally relevant social dialogue, personalized feedback, problem solving, goal-setting and education information based on progress, could upload daily steps from a simple pedometer and data were used to generate tailored feedback and advice, information could be printed out and taken home, information also available in Spanish  CG: Waitlist | - Significant between-group differences in increase of minutes of walk/week of 226.7, 95% *CI* = 107.0, 346.4, *F* (1, 38) = 13.6, *P* = .0008, effect size = 1.2 - IG recorded significant increase in objectively measured daily steps (slope analysis *P* = .002, effect size = .8) post-intervention (CG not evaluated) |
| Kiropoulos et al (2011) [66]  Australia | Ethnic minorities (Greek and Italian Australian)  Depression | Website  Computer | Multicultural Information on Depression online (MIDonline)  IG: Received information about depression and how to find professional care, information also available in Greek and Italian  CG: Semi-structured interview for asking depression questions | - At post-intervention:   IG showed significantly higher depression literacy scores than CG (*F*(1, 178) = 144.99, *P* < .001)  IG showed significantly decreased personal stigma than CG (*F*(1, 178) = 38.75, *P* <.001) but no difference in perceived stigma  No significant difference between groups for depression   - At 1-week follow-up:   IG showed significantly higher depression literacy scores than CG (*F*(1, 178) = 129.13, *P* < .001)  IG showed significantly decreased personal stigma than CG (*F*(1, 178) = 11.08, *P* = .001) but no difference in perceived stigma  No significant difference between groups for depression |
| Lee et al (2014) [67]  Lee et al (2016) [68]  USA | Ethnic minorities (Korean American)  Cervical cancer | Mobile app  Mobile phone | mScreening – Human Papillomavirus (HPV) vaccine/Pap test  Intervention: Received information on cervix and cervical cancer and introduction to HPV vaccine/pap test, information on healthcare accessibility, testimony of women with HPV vaccine/pap test experience and cervical cancer survivors | - At post-intervention:   Significant increase in pap test knowledge (*P* < .001) and HPV knowledge (*P* < .001)  No significant difference in intent to receive the pap test (mean .23, 95% *CI*: -.04 to .51, *P* = .90)  Significant increase in intent to receive HPV vaccine (mean difference = .47, 95% *CI*: .21 to .72, *P* < .001)  3% of participants reported receiving the pap test after intervention and 16.7% of participants reported receiving the first shot of the HPV vaccine   - At 3-month follow-up:   An additional 20% reported receiving the pap test, indicating a total of 23% of participants reported receiving the pap test (95% *CI*: 10% to 42%)  An additional 13.3% of participants reported receiving first shot of the HPV vaccine, indicating that a total of 30% of participants received the HPV vaccine (95% *CI*: 9.9% to 42.3%) |
| Lee et al (2017) [69]  USA | Ethnic minorities (Korean American)  Breast cancer | Mobile app  Mobile phone | mMammogram  IG: Individually tailored and interactive daily messages with information about breast cancer and screening, link to a website with a list of area clinics  CG: Printed information on breast cancer screening | - IG had a significantly higher proportion of participants completed mammograms than CG (75% vs. 30%, *X^2^*_1_ = 24.4, *P* < .001) at 6-month follow-up |
| MacDonell et al (2016) [70]  USA | Ethnic minorities (African American)  Asthma | Website &  text message  Computer & mobile phone | The Detroit Young Adult Asthma Project  IG: Attended 2 computer sessions with information on medication adherence based on Motivational Enhancement System for Adherence, participants to report on medication adherence and asthma control, 30 days of tailored text messages after session 1 and could choose to opt out of text messages after session 2  CG: Access to general online health information | - No significant difference between groups for medication adherence (*P* ≤ 0.05) - IG showed significantly larger magnitude decrease than CG in total symptoms, *t*(42) = 2.22,   *P* < .05 (*d* = .071) and average symptoms per day, *t*(42) = 2.22, *P* < .05 (*d* = .71) |
| Marcus et al (2016) [71]  USA | Ethnic minorities (Latino American)  Physical inactivity | Website & email  Computer | Pasos Hacia La Salud (Steps to Health)  IG: Goal setting, self-monitoring of activities, access to online resources such as maps to plan walking routes and exercise videos, monthly questions to generate tailored PA reports, message board to cultivate social support, 'Ask the expert' feature to ask questions anonymously, weekly email prompts about new updates, information also available in Spanish  CG: Access to general online health information | - IG reported significantly more increased minutes/week of moderate to vigorous PA (MVPA) than CG (mean difference = 50.00, *SE* = 0.95, *P* < 0.01) - IG recorded more objectively measured PA than CG (mean differences = 31.0, *SE* = 10.7, *P* < .01) |
| Mauriello et al (2016) [72]  USA | Ethnic minorities (Latino American) & Low-income groups  Pregnancy | Website  Tablet | Healthy Pregnancy: Step by Step  IG: Used health risk assessment to identify a primary and a secondary risk behavior, setting goals to reduce risk behaviors with feedback and strategies to achieving the goals, feedback were integrated with calculators, quizzes, action plans, support messages, and recipe ideas, printed reports distributed at the conclusion of each session, information also available in Spanish  CG: Access to general printed information | - At post-intervention:   IG self-reported significantly fewer behavior risks than CG (.85 vs 1.20, *OR* = .70, *P* < .001), and more cups of FV per day than CG (4.31 cups vs. 3.32 cups, *OR* = 2.74, *P* < .001)  No significant difference in stress management (*P* = .288), measured by self-report of minutes used daily in stress management activities   - At 4-month postpartum:   IG reported significantly fewer self-report behavior risks than CG (0.72 vs 0.91, OR = 0.81, *P* = .039) and more cups of FV per day than CG (4.43 cups vs. 3.70 cups, OR = 2.16, *P* = .004)  No significant difference in stress management (*P* = .559) |
| Miller et al (2018) [73]  USA | Low-literacy groups & Low-income groups  Colorectal cancer | Mobile app & text message  Tablet and mobile phone | mPATH-CRC (Mobile Patient Technology for Health – Colorectal Cancer)  IG: Provision of information on needs for screening and assisted decision-making, could ‘self-order’ a screening test, automated electronic messages to help complete chosen test  CG: Watched a video about diet and exercise | - IG participants were twice more likely than CG to complete a screening test (30% vs. 15%) (Significance not reported) |
| Moussa et al (2013) [74]  USA | Ethnic minorities (African American)  Diabetes | Website  Computer | eCare We Care  IG: Received information on diabetes, meal planning, eye complications, and foot care, access to credible websites  CG: Access to general printed information | - IG showed significantly higher diabetes literacy scores for all 4 weeks: paired *t* (22) = 5.08, *P* < .001 for week 1; paired *t* (22) = 6.05, *P* < .001 for week 2; paired *t* (22) = 6.74, *P* = .002 for week 3; and paired *t* (22) = 8.92, *P* < .001 for week 4 |
| Neafsey et al (2011) [75]  USA | Older adults  Hypertension | Website  Tablet | PEP-NG (Personal Education Program)  IG: Tailored educational information with printout provided  CG: Access to general online health information | - IG reported significantly decrease of self-medication adverse behavior risk score (paired *t* (73) = 2.17, *P* = .033) (Between group significance not reported) |
| Nelson et al (2016) [76]  USA | Low-income groups  Diabetes | Text message  Mobile phone | MED (Messaging for Diabetes)  Intervention: Daily tailored one-way text messages that addressed barrier to medication adherence, daily two-way text messages to assess adherence performance, personalized adherence feedback with an interactive voice response phone call every week | - No improvement on medication adherence (adjusted odd ratio *AOR* 1.49, 95% *CI*: .66 to 3.10) |
| Neuenschwander et al (2013) [77]  USA | Low-income groups  Nutrition | Website & email  Computer | SNAP-Ed (Supplemental Nutrition Assistance Education Program)  IG: Received educational information on nutrition, goal setting, could use email to ask questions about nutrition  CG: Received in-person education | - No significant difference between groups in nutrition-related behavior outcomes (*P* > .05) |
| Phelan et al (2017) [78]  USA | Low-income groups  Postpartum weight retention | Website & text message  Computer & mobile phone | Fit Moms/ Mamas Activas  IG: Addition to the Supplemental Nutrition Program for Women, Infants, and Children (WIC) program, received weekly lesson, guidance and resources on weight loss, diary and weight and physical activity tracker, message board for support and enquiries, weekly text messages about new updates and feedback, information also available in Spanish  CG: WIC program only | - IG showed significantly greater mean 12-month weight loss than CG (mean difference = 2.3kg, 95% *CI*: 1.1 to 3.5, *P* < .001) |
| Rubinstein et al (2016) [79]  Argentina, Guatemala & Peru | Low-income groups  Pre-hypertension | Text message  Mobile phone | Text message for prehypertension  IG: Weekly personalized text messages containing advice for lifestyle modification, also supported by monthly counselling calls  CG: Usual care with added information on healthy lifestyles | - No significant difference between IG and CG in systolic blood pressure (mean difference = -.37 mm Hg, 95% *CI*: -2.15 to 1.40, *P* = .43) and diastolic blood pressure (mean difference = .01 mm Hg, 95% *CI*: -1.29 to 1.32, *P* = .99) |
| Ryan et al (2013) [80]  USA | Ethnic minorities (African American) & Low-income groups  Diabetes | Website  Computer | HEAT-IT-UP (Health Education and Access Through Information Technology Utilization Program)  Intervention: Received educational and motivational messages, tailored advice based on blood sugar level uploaded, web-access to healthcare providers, peer network | - Significant reduction in HbA1c levels from 7.54% to 6.92% (*t* = 2.24, *df* = 15, *P* = .41) - No significant difference in Low-density lipoprotein (LDL) cholesterol (from 111.08 mm/dL to 95.38 mm/dL, *P* = .104) - No significant difference in High-density lipoprotein (HDL) cholesterol (from 52.54 mm/dL to 48.54 mm/dL, *P* = .178) |
| Steinberg et al (2013) [81]  USA | Ethnic minorities (African American)  Obesity | Text message & email  Mobile phone | Shape Plan  IG: Daily text messages to report on lifestyle behavior and receive tailored feedback and tips, summary of report via email, also supported by 2 face-to-face group sessions  CG: 2 in-person sessions and general education materials | - No significant difference in weight between groups (mean difference = -2.41kg, 95% *CI*: -5.22 to .39, *P* = .09) |
| Tessaro et al (2007) [82]  USA | Low-income groups & Rural communities  Cardio-vascular disease | Website  Computer | Cookin’ Up Health  IG: Received information on fats, serving sizes and nutrition labelling, culturally relevant and individualized intervention, used a cooking show theme to discuss benefits of healthy eating, demonstration of meal preparation  CG: Activity not reported | - No significant difference between groups for fruit and vegetable servings (*P* = .32) and fat intake (*P* = .33), as measured by the 34-item food frequency checklist - IG showed significantly higher dietary knowledge score than CG (*P* = .008) |
| Titov et al (2015) [83]^a^  Australia | Older adults  Depression | Website & email  Computer | Managing Your Mood Course  Aim: To treat depression/ Duration: 8 weeks  Platform:  IG: 5 lessons to learn and practice psychological skills, homework assignment for each lesson, regular reminders and email messages from therapist  CG: Waitlist | - At post-intervention:   IG showed significantly lower depression scores (Cohen's *d* = 2.08; 95% *CI*: 1.38 to 2.72, p < 0.001) and anxiety scores (Cohen's *d* = 1.22; 95% *CI*: .61 to 1.79, *P* < .001) than CG   - At 3-month and 12-month follow-up:   No significant change in anxiety and depression for IG (*P* s > .05) (CG joined program and not assessed) |
| Ünlü Ince et al (2013) [84]  The Netherlands | Ethnic minorities (Turkish Dutch)  Depression | Website & email  Computer | AOC-TR (Alles Onder Controle, AOC: Everything under Control, Turkish version)  IG: 5 weekly sessions presenting the 6-step problem-solving procedure, feedback on homework assignments in weekly email, information also available in Turkish  CG: Waitlist | - At post-intervention:   No significant difference between IG and CG (Cohen's *d* = .37, 95% *CI*: -.03 to .78, *P* = .07) in depression   - At 4-month follow-up:   ITT analysis not undertaken due to high attrition rate (62%) |
| Wahbeh et al (2016) [85]  USA | Older adults  Mood and cognitive function | Website  Tablet or computer | Internet Mindfulness Meditation Intervention  Aim: To promote mood and cognition function/ Duration: 6 weeks  Platform:  IG: Received 6 weekly 1-hour session with 30 minutes of daily home practice, 3-minute meditation which could be practiced with or without guided recording, tips on mindful daily activities, assessment at the end of each session  CG: access to general online health information | - No significant differences between groups on depression (*P* > .32), mindfulness (*P* = .32) and positive and negative feelings (*P* = .32) |
| Wayne et al (2015) [86]  Canada | Low-income groups  Diabetes | Mobile app  Mobile phone | Health Coaching with Connected Wellness Platform  IG: Access to Connected Wellness Platform which allowed for tracking of blood glucose level, physical activity and food consumption, guidance to healthy lifestyle choices based on health goals and routines, could contact health coach through the app anytime  CG: Face-to-face health coaching | - No significant differences between groups on HbA1c (intention to treat: *P* = .48, per-protocol: *P* = .83) |

^a^Adaptations of similar program
