# Supplementary material for: Applying the Electronic Health Literacy Lens: Systematic Review of Electronic Health Interventions Targeted at Socially Disadvantaged Groups
Source: J Med Internet Res. 2020 Aug 13;22(8):e18476. doi: 10.2196/18476 (PMC7453328; doi:10.2196/18476)
Supplement: Multimedia Appendix 4 [file jmir_v22i8e18476_app4.docx]

**Multimedia Appendix 4: Summary of eHealth literacy domains likely addressed**

| Authors (Year) | Target group(s) | Intervention components |
| --- | --- | --- |
| **1. Using technology to process health information** | | |
| Agyapong et al (2017) [37] | Rural communities | - Information about mood and depression |
| Anand et al (2016) [38] | Ethnic minorities | - Culturally relevant health messages - Information written at grade 7 level |
| Arora et al (2014) [39] | Low-income groups & Ethnic minorities | - Educational and motivational messages - Messages written at grade 5 level - Trivia messages to increase knowledge of diabetes |
| Bennett et al (2018) [40] | Low-income groups | - Skills training materials available in print and video formats - Sharing information with health professionals |
| Bond et al (2010) [41] | Older adults | - Instructions on disease management, diet and exercise - Sharing information with study nurse and other participants - Recording of self-management activities such as blood glucose level, medication administration and related activities - Access to sites on diabetes and other related topics |
| Broekhuizen et al (2016) [42] & Wijsman et al (2013) [43] | Older adults | - General recommendations on physical activity (PA) - Daily recording of activities |
| Buller et al (2008) [44] | Rural communities | - Content of health benefits of fruit and vegetable (FV) - Information presented in different formats - Locally relevant information - Access to other health resources |
| Carroll et al (2019) [45] | Low-income groups & Low-literacy groups & Ethnic minorities | - Information on common HIV medications with pill pictures - Information about lab tests with brief and understandable explanations |
| Caster et al (2017) [46] | Rural communities | - Information about cervical cancer and screening presented in a culturally appropriate way - Information presented in video and audio formats |
| Chen et al (2016) [47] | Older adults | - Relaxation guiding practice presented in multimedia formats - A digital sleep diary for recording sleep patterns - Sleep information presented in simple graphical illustrations |
| Chen et al (2018) [48] | Low-income groups & Rural communities | - Information about diabetic retinopathy and the need for eye examination |
| Choi et al (2012) [49]^a^ | Ethnic Minorities | - Online lessons on depression - Resources about communication skills, anxiety, and strategies for improving sleep - Culturally relevant information - Use of illustrated stories |
| Dear et al (2015) [51]^a^ | Older adults | - Lessons to learn and practice psychological skills - Information presented in images and age-appropriate case studies - Additional resources such as assertiveness, communication and problem-solving skills available |
| Fortmann et al (2017) [53] | Ethnic minorities | - Culturally appropriate educational messages |
| Gilmore et al (2017) [54] | Low-income groups | - 16 SmartTips sent with health information on diet, physical activity, sleep and lifestyle - Weight and steps displayed as graph |
| Griffin et al (2018) [55] | Low-income groups | - Text messages with education on diet and exercise - Weblinks to other resources |
| Hacking et al (2016) [56] | Low-income groups | - Information about hypertension and healthy lifestyle suggestions |
| Hageman et al (2014) [57] | Rural communities | - Instructions about healthy eating and blood pressure reduction activities - Information presented as visual display and video demonstrations - Recording of blood pressure and step counts |
| Herring et al (2017) [58] | Ethnic minorities | - Text messages about behavioral goals targeting at self-weighing, energy intake and physical activity - Simple messages suitable for participants with low literacy - Weekly Facebook posts providing links to websites and videos about skills training |
| Hill et al (2006) [59] & Weinert et al (2008) [87] | Rural communities | - Teaching units with a wide variety of materials and topics such as accessing health information online |
| Hong et al (2015) [60] | Older adults | - Information on healthy living - Links to reliable health information - Interactive calendar to record activities and log the number of minutes exercised - Could view progress as bars, lines, and/or as a calendar |
| Jarvis et al (2019) [62] | Older adults | - Key psycho-education messages on factors underlying loneliness delivered through WhatsApp after face-to-face sessions |
| Joseph et al (2015) [63] | Ethnic minorities | - Weekly physical activity (PA) promotion materials on Facebook wall - Culturally relevant information - Information written at grade 10 reading level or lower - Sharing personal experiences with other participants - Recording of daily step counts |
| Kamal et al (2015) [64] | Low-income groups | - Health information text messages |
| King et al (2013) [65] | Ethnic minorities & Low-income groups | - Educational information presented as simple speech, hand gesture or facial cues - Conversation written at less than grade 8 level |
| Kiropoulos et al (2011) [66] | Ethnic minorities | - Information about depression, written at grade 7 or 8 level - Culturally relevant materials - Case studies used - Information to find a bilingual mental health professional and care |
| Lee et al (2014) [67] & Lee et al (2016) [68] | Ethnic minorities | - Health information about cervical cancer and HPV vaccine - Culturally relevant testimonies from peers about cervical cancer diagnosis and experiences with HPV vaccination - Offer information of clinics near each participant |
| Lee et al (2017) [69] | Ethnic minorities | - Health information about breast cancer and screening and addressed cultural barriers - Link to website about local clinics - Messages included illustrations, video clips and photos - Embedded GPS to determine the distance and direction of clinics - Prompts to trigger action to make appointments - Presented stories of other Korean American women about their screening experiences |
| MacDonell et al (2016) [70] | Ethnic minorities | - Small amount of psycho-education with motivational elements - Presented by animated characters - Information about decisional balance - Reporting of symptoms, affect, behavior and cognition in real time |
| Marcus et al (2016) [71] | Ethnic minorities | - Culturally adapted information about physical activity (PA) - Information presented in video and graph formats - Reporting of PA level - Message board to share information - Links to other online and community resources |
| Mauriello et al (2016) [72] | Ethnic minorities | - Information written at grade 4 or 5 reading level - Culturally relevant information |
| Miller et al (2018) [73] | Low-literacy groups & Low-income groups | - 8.6 minutes video about Colorectal cancer (CRC) screening decision - Information written at grade 6 reading level |
| Moussa et al (2013) [74] | Ethnic minorities | - Information about diabetes presented in simple language and graphics - Culturally relevant materials - Access to credible health information websites |
| Neafsey et al (2011) [75] | Older adults | - Time of medication presented as animated clock - Reporting of symptoms and medication use |
| Nelson et al (2016) [76] | Low-income groups | - Daily one-way text messages about medication adherence barriers - Reporting of medication adherence by daily two-way text messages |
| Neuenschwander et al (2013) [77] | Low-income groups | - Information about nutrition presented in various learning styles and video clips |
| Phelan et al (2017) [78] | Low-income groups | - Lessons about weight loss - Information presented in short content, pictures and videos - Instructional and inspirational videos - Diary to record activities - Links to related sites |
| Rubinstein et al (2016) [79] | Low-income groups | - Content culturally adapted for each country |
| Ryan et al (2013) [80] | Ethnic minorities | - Educational messages were culturally relevant and easy to read for individuals with limited education - Web-based access to healthcare providers - Reporting of blood sugar level every morning |
| Steinberg et al (2013) [81] | Ethnic minorities | - Reporting of performance on goals from the previous day - Graphs of progress |
| Tessaro et al (2007) [82] | Low-income groups & Rural communities | - Culturally targeted information about healthy eating - All information audio-based with video and pictorial animation - Key audio points available as text |
| Titov et al (2015) [83]^a^ | Older adults | - Online lessons about depression - Information available in text and images - Case studies presented - Additional resources such as assertiveness communication skills, problem solving available |
| Ünlü Ince et al (2013) [84] | Ethnic minorities | - Culturally relevant information about symptoms of depression - Recognizable examples |
| Wahbeh et al (2016) [85] | Older adults | - Instructions and discussion on stress, meditation, relaxation and mind-body interaction - Information about various forms of practice |
| Wayne et al (2015) [86] | Low-income groups | - Monitor key metrics such as blood glucose, mood, physical activity and food consumption - Use of graphics and pictures |
| **2. Understanding of health concepts and language** | | |
| Anand et al (2016) [38] | Ethnic minorities | - Bi-weekly health messages and brief weekly email or text tailored to individual participant's stage of change - Advice and support geared to motivate participants to make changes |
| Arora et al (2014) [39] | Low-income groups & Ethnic minorities | - Medication reminders - Messages that offered participants with a concrete and attainable goal of the day |
| Bennett et al (2018) [40] | Low-income groups | - Self-monitoring four behavior change goals each week using interactive voice response or text messaging - Self-weighting daily using a cellular connected scale - Algorithms changed participant's assigned goals bimonthly to prevent habituation and promote novelty |
| Bond et al (2010) [41] | Older adults | - Developing personal action plans - Tailored instruction |
| Broekhuizen et al (2016) [42] & Wijsman et al (2013) [43] | Older adults | - Self-monitoring of activities by an accelerometer - Setting goals to increase PA and had control to adjust the goals |
| Buller et al (2008) [44] | Rural communities | - Practical skills and recipes |
| Carroll et al (2019) [45] | Low-income groups & Low-literacy groups & Ethnic minorities | - Set up reminders for appointments and taking and refilling medications - Personalized information - Personalized list of potential questions to ask clinician |
| Caster et al (2017) [46] | Rural communities | - Quiz to engage participants in learning |
| Chen et al (2016) [47] | Older adults | - Assignments of daily activities and practices - Reminders to adhere to prescribed daily activities |
| Choi et al (2012) [49]^a^ | Ethnic Minorities | - Homework assignments |
| Dang et al (2017) [50] | Ethnic minorities | - Daily monitoring of body weight - Answering of 10 daily questions |
| Dear et al (2015) [51]^a^ | Older adults | - Homework assignments |
| Dugas et al (2018) [52] | Older adults | - Fitbit One provided for easy self-monitoring - Earn points by achieving better self-care goals |
| Fortmann et al (2017) [53] | Ethnic minorities | - Motivational messages, prompts for medication reminders and blood glucose monitoring - Encourage participants to text in their next observed value of blood glucose level |
| Gilmore et al (2017) [54] | Low-income groups | - BodyTrace scale and an accelerometer for self-monitoring - The weight graph included an individualized postpartum weight loss zone - Individual step count goal - Short interactive activity to assess understanding of materials |
| Griffin et al (2018) [55] | Low-income groups | - Using a scale and pedometer for self-monitoring - Received goal-setting prompts that required a response - Received reminders or questions about healthy eating or exercise - eNewsletter with reminders, tips, and a low-cost healthy recipe - Prompts to measure body weight every week |
| Hacking et al (2016) [56] | Low-income groups | - Practical health tips on diet and exercise |
| Hageman et al (2014) [57] | Rural communities | - Tailored behavioral newsletters and messages - Self-assessment quizzes - Blood pressure monitor and a pedometer provided for self-monitoring |
| Herring et al (2017) [58] | Ethnic minorities | - Daily text messages tailored to each behavioral goal - Digital scale, pedometer, water bottle and portion plate provided for self-monitoring - Self-monitoring texts sent 3 to 4 times weekly to probe behavioral adherence |
| Hill et al (2006) [59] & Weinert et al (2008) [87] | Rural communities | - Access to 'Health Roundtable', an expert-facilitated chat room for discussions related to the teaching units |
| Hong et al (2015) [60] | Older adults | - Cued to set up long-term goal and weekly short-term goal - Examples of goals provided to guide goal setting - Activity tracking - Progress reviews |
| Ingersoll et al (2015) [61] | Rural communities | - Daily queries about medication adherence (once), mood (twice) and substance use (once) |
| Jarvis et al (2019) [62] | Older adults | - Homework assignments requiring a WhatsApp response - Individualized messages on how to counter maladaptive cognitions |
| Joseph et al (2015) [63] | Ethnic minorities | - Pedometer provided for self-monitoring - Setting goals - Text messages with tips and strategies to increase PA - Individual adaptive step goals from study staff via email |
| Kamal et al (2015) [64] | Low-income groups | - Reminders tailored to individual prescriptions - Responses required to state if medication was taken - Health information tailored to medical and drug profile of individual participant |
| King et al (2013) [65] | Ethnic minorities & Low-income groups | - Pedometer provided for self-monitoring - Individualized social dialogue - Setting goals - Problem solving skills - Information could be downloaded and printed |
| Lee et al (2014) [67] & Lee et al (2016) [68] | Ethnic minorities | - Questions and quizzes to test knowledge - Individually tailored messages |
| Lee et al (2017) [69] | Ethnic minorities | - Individually tailored messages - Responses to some messages required - Questions to test knowledge |
| MacDonell et al (2016) [70] | Ethnic minorities | - Setting goals - Tailored information |
| Marcus et al (2016) [71] | Ethnic minorities | - Setting goals - Self-monitoring of activities - Tailored manual |
| Mauriello et al (2018) [72] | Ethnic minorities | - Tailored program based on stages of change - Selection of goals and action plans - Quizzes and recipes ideas available - Feedback could be printed |
| Miller et al (2018) [73] | Low-literacy groups & Low-income groups | - CRC screening test request available - Could sign up for follow-up text messages to support the screening process |
| Moussa et al (2013) [74] | Ethnic minorities | - Diabetes knowledge survey at the end of each session |
| Neafsey et al (2011) [75] | Older adults | - Tailored educational content - Reported symptoms, medication use and corrective strategies available in printed versions |
| Nelson et al (2016) [76] | Low-income groups | - Motivational messages - Problem solving questions to enhance future adherence success through interactive voice response (IVR) calls |
| Neuenschwander et al (2013) [77] | Low-income groups | - Setting goals - Interactive activities - Downloadable recipes |
| Phelan et al (2017) [78] | Low-income groups | - Setting goals - Weight and physical activity tracker - Self-monitoring of activities |
| Rubinstein et al (2016) [79] | Low-income groups | - Customized text messages based on participant's stage of change - Text messages with practical suggestions |
| Ryan et al (2013) [80] | Ethnic minorities | - Glucometer and test strips provided for self-monitoring - Chat application to allow the participants to engage in live, web-based chats with registered nurse - Downloadable educational materials |
| Steinberg et al (2013) [81] | Ethnic minorities | - Pedometer provided for self-monitoring - Self-monitoring of performance on goals - Prompts to improve adherence |
| Tessaro et al (2007) [82] | Low-income groups & Rural communities | - Used a cooking theme to engage with cooking demonstration - Recipes and tips on nutrition - Tailored recipes to watch based on screening questions |
| Titov et al (2015) [83]^a^ | Older adults | - Homework assignments |
| Ünlü Ince et al (2013) [84] | Ethnic minorities | - Homework assignments - Problem solving procedures to deal with problems |
| Wahbeh et al (2016) [85] | Older adults | - Skills to modify stress reactions - Daily practice and tips |
| Wayne et al (2015) [86] | Low-income groups | - Guidance on healthy choices from health coach based on uploaded health goals and routines - Goal setting and progress monitoring |
| **3. Ability to actively engage with digital services** | | |
| Bond et al (2010) [41] | Older adults | - Training to use the website |
| Buller et al (2008) [44] | Rural communities | - One-on-one computer skills training - Freely navigational node structure to engage users |
| Carroll et al (2019) [45] | Low-income groups & Low-literacy groups & Ethnic minorities | - Provision of 6 x 90-minute training sessions - An individual coaching session to reinforce skills learned |
| Caster et al (2017) [46] | Rural communities | - Short video to show how to use the tablet and interact with the screen - Easy navigation by touching the screen with finger or a stylus - Staff available to assist in using the tablet |
| Dugas et al (2018) [52] | Older adults | - Group training session to use devices at the start of the intervention |
| Fortmann et al (2017) [53] | Ethnic minorities | - Instructions on how to send and receive text messages |
| Hageman et al (2014) [57] | Rural communities | - Training session on using the website - Instructions to use the blood pressure monitor and pedometer provided |
| Hill et al (2006) [59] & Weinert et al (2008) [87] | Rural communities | - Training session on the use of the system |
| Hong et al (2015) [60] | Older adults | - Instruction videos on how to use iCanfit available on the 'Help' page |
| Jarvis et al (2019) [62] | Older adults | - 4 x 90-minute face-to-face group training sessions - Weekly Help Desk for technical support |
| Kamal et al (2015) [64] | Low-income groups | - Demonstration by sending one test SMS on participant's mobile phone |
| King et al (2013) [65] | Ethnic minorities & Low-income groups | - Training provided |
| Marcus et al (2016) [71] | Ethnic minorities | - Training to use the website provided |
| Miller et al (2018) [73] | Low-literacy groups & Low-income groups | - Touch screen interface for easy navigation - Narrator available to guide through the program |
| Moussa et al (2013) [74] | Ethnic minorities | - Voice prompt to assist navigation - Navigational guidance using text and arrows |
| Neafsey et al (2011) [75] | Older adults | - Touchscreen interface to allow for easy navigation |
| Ryan et al (2013) [80] | Ethnic minorities | - Training to use the intervention at the start and periodic refresher training provided - Provided training guide on how to upload blood sugar level data |
| Tessaro et al (2007) [82] | Low-income groups & Rural communities | - Touch screen to allow for easy navigation |
| Wahbeh et al (2016) [85] | Older adults | - Training provided |
| **4. Feel safe and in control** | | |
| Bond et al (2010) [41] | Older adults | - Log in required |
| Broekhuizen et al (2016) [42] & Wijsman et al (2013) [43] | Older adults | - Secure database and personal website |
| Carroll et al (2019) [45] | Low-income groups & Low-literacy groups & Ethnic minorities | - Password protected |
| Chen et al (2016) [47] | Older adults | - Log in required |
| Choi et al (2012) [49]^a^ | Ethnic Minorities | - Secure messaging |
| Dang et al (2017) [50] | Ethnic minorities | - Identifiable information not stored on the mobile phone - Data could be viewed only by the study coordinator on a secure website |
| Dear et al (2015) [51]^a^ | Older adults | - Secure email and messaging |
| Gilmore et al (2017) [54] | Low-income groups | - Individual IDs and passwords required |
| Hageman et al (2014) [57] | Rural communities | - Password-protected |
| Herring et al (2017) [58] | Ethnic minorities | - Facebook group by invitation only - Information about privacy settings provided |
| Hill et al (2006) [59] & Weinert et al (2008) [87] | Rural communities | - Log in required - Private email access to other participants and nurse |
| Hong et al (2015) [60] | Older adults | - Username and password required |
| Ingersoll et al (2015) [61] | Rural communities | - Queries on substance use masked as a weather question |
| Jarvis et al (2019) [62] | Older adults | - Closed WhatsApp group |
| Joseph et al (2015) [63] | Ethnic minorities | - Facebook was 'closed', only participants could view information |
| King et al (2013) [65] | Ethnic minorities & Low-income groups | - Personal ID - Headphone to hear conversation for privacy |
| Marcus et al (2016) [71] | Ethnic minorities | - Could ask questions anonymously |
| Phelan et al (2017) [78] | Low-income groups | - Username and password required |
| Ryan et al (2013) [80] | Ethnic minorities | - Log in required |
| Titov et al (2015) [83]^a^ | Older adults | - Secure email and messaging |
| Ünlü Ince et al (2013) [84] | Ethnic minorities | - Username and password required |
| Wahbeh et al (2016) [85] | Older adults | - Log in required |
| Wayne et al (2015) [86] | Low-income groups | - Secure messaging - Health data visible to health coach through a secure web portal - Two-way certificate-based authentication - Passwords stored in encrypted columns |
| **5. Motivated to engage with digital services** | | |
| Agyapong et al (2017) [37] | Rural communities | - Text messages sent twice daily - New messages with no repetition |
| Anand et al (2016) [38] | Ethnic minorities | - Peer to peer support and motivational sessions available in monthly Skype webinar with experts and for peer and social support among participants |
| Arora et al (2014) [39] | Low-income groups & Ethnic minorities | - Messages sent out in a regular basis |
| Bennett et al (2018) [40] | Low-income groups | - Personalized feedback message with a short skills training tip, based on the participant's weight loss progress - Retry protocol activated if participants did not answer scheduled counselling calls |
| Bond et al (2010) [41] | Older adults | - Tailored feedback - Weekly discussion group to receive education material and facilitate peer support - Access to study nurse and other participants through email and instant messaging for support |
| Broekhuizen et al (2016) [42] & Wijsman et al (2013) [43] | Older adults | - Regular updates of PA status by email - Regular feedbacks based on data from accelerometer - Easy communication through emails - Personal guidance available |
| Buller et al (2008) [44] | Rural communities | - Email updates to encourage return to the website - Regular new content |
| Chen et al (2016) [47] | Older adults | - Immediate feedback |
| Choi et al (2012) [49]^a^ | Ethnic Minorities | - Regular reminder and notification emails |
| Dang et al (2017) [50] | Ethnic minorities | - 3 messages 15 minutes apart if no response to the first automated message |
| Dear et al (2015) [51]^a^ | Older adults | - Regular email reminder and notifications |
| Dugas et al (2018) [52] | Older adults | - Viewed scores to see if they were trending at, below or above point goals for each behavior - Interacted with peers or clinician online for some groups |
| Fortmann et al (2017) [53] | Ethnic minorities | - 2 to 3 messages sent a day but frequency tapering over 6 months |
| Gilmore et al (2017) [54] | Low-income groups | - Personalized advice if body weights above or below the zone for 3 consecutive days via phone, email or text messages |
| Griffin et al (2018) [55] | Low-income groups | - 2 - 3 short identical messages daily - eNewsletters sent weekly |
| Hacking et al (2016) [56] | Low-income groups | - 90 messages disseminated over 17 weeks |
| Hageman et al (2014) [57] | Rural communities | - Newsletters and messages sent regularly |
| Herring et al (2017) [58] | Ethnic minorities | - Morning text message prompts with immediate personalized automatic feedback to reinforce successes and/or provide support - Received raffle entries if responded to self-monitoring text prompts - Facebook group for support |
| Hill et al (2006) [59] & Weinert et al (2008) [87] | Rural communities | - Bi-weekly chat sessions with new health-related topics initiated by nurse monitor - Access to 'Koffee Klatch' chatroom to have conversations with other participants to share life experiences, express concerns and provide support |
| Hong et al (2015) [60] | Older adults | - Regular updates - Personalized feedbacks based on information entered - Online network to connect users |
| Ingersoll et al (2015) [61] | Rural communities | - Tailored personalized response based on replies to messages |
| Joseph et al (2015) [63] | Ethnic minorities | - Discussion prompts to encourage dialogue on group Facebook - Gave and received social support through Facebook - Text messages to remind participants of weekly new topics |
| Kamal et al (2015) [64] | Low-income groups | - Regular reminders |
| King et al (2013) [65] | Ethnic minorities & Low-income groups | - Personalized feedback based on pedometer data and progress review |
| Lee et al (2017) [69] | Ethnic minorities | - Earned a digital pink ribbon for responses |
| MacDonell et al (2016) [70] | Ethnic minorities | - Tailored feedback based on responses |
| Marcus et al (2016) [71] | Ethnic minorities | - Email prompts and phone calls to access the website - Regular new information - Tailored feedback |
| Mauriello et al (2016) [72] | Ethnic minorities | - Tailored feedback and strategies to achieve the selected goal - Support messages |
| Nelson et al (2016) [76] | Low-income groups | - Weekly IVR calls with personalized feedback in respond to the two-way text messages |
| Neuenschwander et al (2013) [77] | Low-income groups | - Contact us section to ask nutrition questions |
| Phelan et al (2017) [78] | Low-income groups | - Weekly text messages to notify participants of new content - Tailored feedback - Message board for support and problem solving |
| Rubinstein et al (2016) [79] | Low-income groups | - Text messages sent weekly |
| Ryan et al (2013) [80] | Ethnic minorities | - Tailored educational and motivational feedback based on blood sugar level data uploaded - Discussion boards - Access to peer network - New diabetes self-management campaign every 3 months |
| Steinberg et al (2013) [81] | Ethnic minorities | - New goals at 3 months to main motivation - Tailored feedback based on reports |
| Titov et al (2015) [83]^a^ | Older adults | - Email reminder and notifications |
| Ünlü Ince et al (2013) [84] | Ethnic minorities | - Feedback on homework in weekly emails |
| Wayne et al (2015) [86] | Low-income groups | - Communicate with health coach anytime to schedule phone or in-person contact |
| **6. Access to digital services that work** | | |
| Arora et al (2014) [39] | Low-income groups & Ethnic minorities | - Provided a free unlimited text messaging plan |
| Bennett et al (2018) [40] | Low-income groups | - Cellular scale connected to the app |
| Broekhuizen et al (2016) [42] & Wijsman et al (2013) [43] | Older adults | - Accelerometer could upload PA data to a secure database and opens personal website |
| Buller et al (2008) [44] | Rural communities | - List of local public computer access sites |
| Dang et al (2017) [50] | Ethnic minorities | - Received a mobile phone - Free data use and 30 free minutes of calling per month |
| Dugas et al (2018) [52] | Older adults | - Tablet with data plan provided |
| Fortmann et al (2017) [53] | Ethnic minorities | - Mobile phone provided - Additional costs for texting provided |
| Gilmore et al (2017) [54] | Low-income groups | - Smartloss app and iPhone provided - Scale and accelerometer wirelessly connected to app |
| Hageman et al (2014) [57] | Rural communities | - Contact information available for technical assistance |
| Hill et al (2006) [59] & Weinert et al (2008) [87] | Rural communities | - Computer and software provided - Available 24 hours a day, 7 days a week - Toll free number available for technical assistance |
| Ingersoll et al (2015) [61] | Rural communities | - Mobile phone provided |
| Jarvis et al (2019) [62] | Older adults | - Smartphone and data package provided |
| Kamal et al (2015) [64] | Low-income groups | - Provided cost of sending text messages with prepaid credit |
| King et al (2013) [65] | Ethnic minorities & Low-income groups | - Used computer at community center - Pedometer data downloaded to computer - Technical assistance from staff available |
| Kiropoulos et al (2011) [66] | Ethnic minorities | - Access to computer provided |
| Lee et al (2017) [69] | Ethnic minorities | - Mobile phone provided - Reimbursement of text message data fees |
| Mauriello et al (2016) [72] | Ethnic minorities | - iPad provided |
| Miller et al (2018) [73] | Low-literacy groups & Low-income groups | - iPad provided |
| Moussa et al (2013) [74] | Ethnic minorities | - Access the program at health centers |
| Neafsey et al (2011) [75] | Older adults | - Access program at health clinics - Technical assistance from staff available |
| Neuenschwander et al (2013) [77] | Low-income groups | - Contact us section for website problems |
| Phelan et al (2017) [78] | Low-income groups | - Accessed from anywhere with internet connection - Technical number for support |
| Ryan et al (2013) [80] | Ethnic minorities | - Desktop computer and internet connectivity provided - Glucometer data could be uploaded to website |
| Tessaro et al (2007) [82] | Low-income groups & Rural communities | - Viewed the program at clinics and could come back if interested - Internet access not required |
| Wahbeh et al (2016) [85] | Older adults | - Access to iPad or own computer - Phone numbers to contact for technical assistance |
| Wayne et al (2015) [86] | Low-income groups | - Provision of a smartphone with data plan - Access to Connected Wellness Platform |
| **7. Digital services that suit individual needs** | | |
| Anand et al (2016) [38] | Ethnic minorities | - Available in Hindi or Punjabi |
| Arora et al (2014) [39] | Low-income groups & Ethnic minorities | - Available in Spanish |
| Caster et al (2017) [46] | Rural communities | - Available in English or Chichewa |
| Choi et al (2012) [49]^a^ | Ethnic Minorities | - Available in English or Chinese |
| Dang et al (2017) [50] | Ethnic minorities | - Available in English or Spanish |
| Hacking et al (2016) [56] | Low-income groups | - Available in Xhosa |
| Kamal et al (2015) [64] | Low-income groups | - Available in participants' preferred language - Bolo SMS (Verbal SMS) option available |
| King et al (2013) [65] | Ethnic minorities & Low-income groups | - Simple touching of responses on computer screen - Available in Spanish or English |
| Kiropoulos et al (2011) [66] | Ethnic minorities | - Available in Greek, Italian or English |
| Lee et al (2017) [69] | Ethnic minorities | - Available in Korean |
| Marcus et al (2016) [71] | Ethnic minorities | - Available in Spanish |
| Mauriello et al (2016) [72] | Ethnic minorities | - Available in English or Spanish |
| Miller et al (2018) [73] | Low-literacy groups & Low-income groups | - Large intuitive response buttons |
| Moussa et al (2013) [74] | Ethnic minorities | - Audio option available - Design elements to assist eHealth use by individuals with low health literacy such as clearly labelled function buttons |
| Neafsey et al (2011) [75] | Older adults | - Large objects (3cm high) and 20-point text size - Adaptive, wide-scroll bars and dropdown menus |
| Phelan et al (2017) [78] | Low-income groups | - Available in English or Spanish |
| Tessaro et al (2007) [82] | Low-income groups & Rural communities | - All instructions when using the program were audio based |
| Ünlü Ince et al (2013) [84] | Ethnic minorities | - Available in Turkish |

^a^Adaptations of a similar intervention.
